# Accelerating Phosphate Transport Across Membranes via Synergistic Chemical‐Electrochemical Carriers

**DOI:** 10.1002/anie.4185923

**Published:** 2026-08-27

**Authors:** Lei Xia, Yangbo Qiu, Qingzhi Liu, Mengjiao Guan, Xi Zhang, Raf Dewil, Jin Shang, Yan Zhao, Bart Van der Bruggen, Chuyang Tang

**Affiliations:** ^1^ Department of Civil Engineering The University of Hong Kong Pokfulam SAR Hong Kong; ^2^ Division of Soil and Water Management KU Leuven Leuven Belgium; ^3^ College of Chemistry and Pharmaceutical Sciences Qingdao Agricultural University Qingdao China; ^4^ College of Chemistry and Chemical Engineering Ocean University of China Qingdao China; ^5^ Pritzker School of Molecular Engineering University of Chicago Chicago Illinois USA; ^6^ Department of Chemical Engineering KU Leuven Process and Environmental Technology Lab Sint‐Katelijne‐Waver Belgium; ^7^ Department of Engineering Science University of Oxford Oxford UK; ^8^ School of Energy and Environment City University of Hong Kong Kowloon Hong Kong; ^9^ Department of Chemical Engineering KU Leuven Leuven Belgium

**Keywords:** carrier‐to‐carrier hopping, electro‐driven carrier‐conducting membrane, membrane crystallization, monodispersed ferrihydrite, phosphate recovery

## Abstract

Efficient phosphate recovery from phosphate‐containing wastewater presents a dual opportunity to mitigate environmental pollution and secure nutrient supply for agriculture. Among many recovery methods, electro‐membrane crystallization (e‐MC) is a promising strategy for sustainable phosphate recovery, yet the low phosphate throughput of conventional electro‐driven membranes constrains its efficacy. Here, we propose an ion carrier‐to‐carrier hopping transport principle and introduce a nanostructured electro‐driven carrier‐conducting membrane (e‐CCM) engineered with monodispersed electro‐ferrihydrite nanoparticles as a built‐in phosphate carrier. This membrane architecture establishes a coordination environment where ≡FeOH acts as transient phosphate binding sites, and the applied electric field promotes directional phosphate migration, thereby accelerating phosphate permeation. Operated at 5 mA cm^−2^, the resulting e‐CCM membrane achieves a phosphate permeation rate of 0.92 mol m^−2^ h^−1^ and a recovery rate of 98.8%, outperforming state‐of‐the‐art ion exchange membranes. We further demonstrate scalability by integrating the e‐CCM membrane in e‐MC for processing simulated urine, achieving 93.6% phosphate recovery and the precipitation of high‐purity struvite. This work pioneers a hypothesis of carrier‐conducting construction that synergizes membrane electrochemical transport with chemical affinity, establishing a scalable and energy‐efficient pathway to close the phosphorus loop and advance circular resource economies.

## Graphical abstract

A new membrane design principle based on synergistic chemical and electrochemical carriers is proposed to construct an electro‐driven carrier‐conducting membrane for high‐throughput phosphate transport. Monodispersed ferrihydrite nanoparticles embedded within the membrane act as built‐in phosphate carriers, enabling accelerated phosphate migration via carrier‐conducting diffusion and carrier‐to‐carrier hopping under an applied electric field.

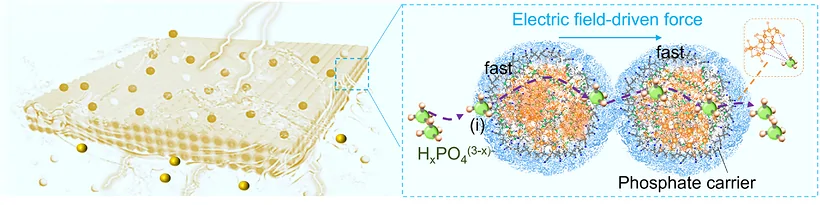

## 1 Introduction

Phosphorus is an essential nutrient for global food production, yet its supply faces mounting scarcity and environmental challenges due to intensive phosphate rock mining (Figure S1) [1, 2]. Global phosphate reserves are geographically concentrated, with 68% in Morocco, and exhibit substantial variability in ore grade and impurity levels (Figure S2a), resulting in fluctuating market prices and declining resource quality [3, 4]. Currently, more than 220 million metric tons of phosphate rock are mined annually, of which approximately 85% is processed into fertilizer [5]. However, excessive and improper fertilizer use results in widespread phosphate pollution and eutrophication (Figure S1) [6, 7]. Data from the Global Freshwater Quality Database (GEMStat) indicates that 48% of 3392 lake sampling stations exhibit long‐term average total phosphate concentrations exceeding 100 µg L^−1^, indicating hypereutrophic status (Figure S2b) [8]. Closing the phosphorus cycle through recovery from waste streams offers a sustainable strategy to mitigate eutrophication and alleviate resource scarcity [9, 10]. Phosphate‐containing waste streams arise from diverse sources, including municipal wastewater, industrial processes, agricultural runoff, and food‐related production, providing substantial opportunities for phosphorus recovery and recycling [11]. Among these sources, urine represents a particularly concentrated phosphorus waste stream. For example, phosphorus recovery from urine alone could offset up to 20% of global phosphorus demand [12, 13].

Conventional approaches for managing phosphate in waste streams, such as adsorption and chemical precipitation, primarily focus on removal rather than recovery [14, 15, 16, 17]. Current recycling practices often rely on the land application of sludge biosolids as fertilizers, but these products frequently suffer from low purity and the presence of hazardous contaminants, limiting their agricultural value [18]. Electrodialysis, utilizing electro‐driven membranes, has emerged as a sustainable and efficient technology for ion extraction [19, 20, 21, 22]. Specifically, recent advancements have integrated electrodialysis with crystallization, developing electro‐driven membrane crystallization (e‐MC) (Figure 1a), which enables the production of high‐purity salts [23, 24, 25]. In e‐MC, an applied electric field drives continuous and high‐throughput ion electromigration across ion exchange membranes, rapidly concentrating target ions to levels required for downstream crystallization. Importantly, the functional objective of e‐MC differs from that of conventional electrodialysis or permselective membrane separations. Rather than maximizing ion selectivity, e‐MC prioritizes fast and sustained ion transport to achieve high productivity and efficient crystallization. Accordingly, membranes employed in e‐MC do not necessarily require high permselectivity but instead must exhibit high permeation rates. Despite the strong potential of e‐MC for simultaneous phosphate recovery and fertilizer production, existing electro‐driven membranes in e‐MC suffer from low phosphate throughputs [24, 26]. This is because phosphate experiences steric hindrance within microporous cavities and faces strong electrostatic repulsion due to its unique tetrahedral geometry and high negative valence [27, 28]. Therefore, tailoring membrane materials that enable fast phosphate transport is critical for realizing fit‐for‐purpose e‐MC technologies.

**FIGURE 1:**
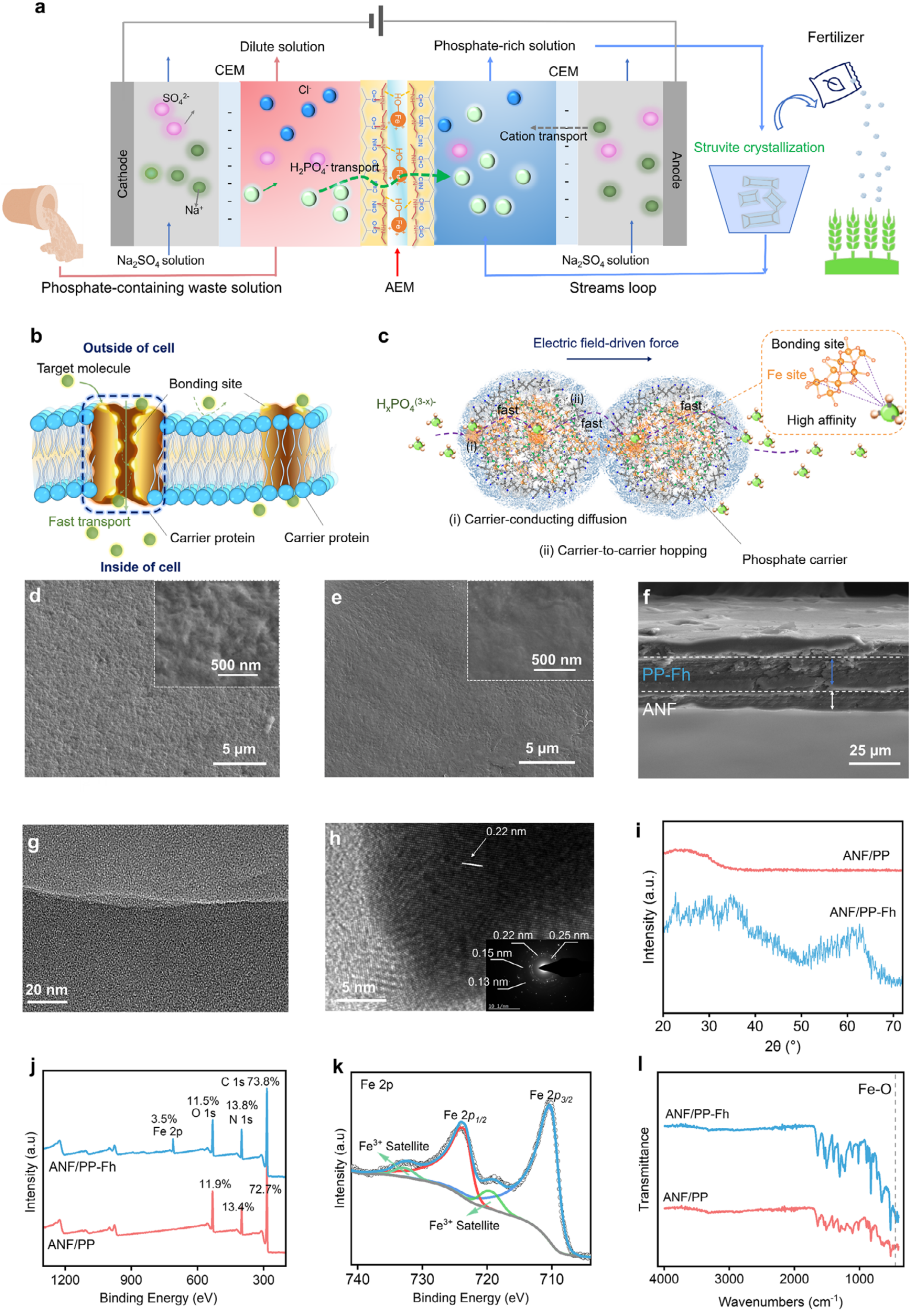
Fabrication and characterization of e‐CCM for phosphate extraction. (a) Electro‐driven membrane crystallization as a sustainable strategy for phosphate extraction from waste streams to close the phosphorus loop. (b) Carrier proteins in cell membranes enable molecule fast transport through specific binding; (c) Concept of carrier‐conducting phosphate transport in engineered systems; (d and e) the surface SEM images of ANF/PP and ANF/PP‐Fh; (f) the cross‐section image of ANF/PP‐Fh; (g) TEM image, (h) HRTEM image, and SAED pattern (inset) of ANF/PP‐Fh; (i) XRD patterns of ANF/PP and ANF/PP‐Fh, confirming the presence of 2‐Line Fh; (j) XPS spectra of the surface elements (atomic%) of ANF/PP and ANF/PP‐Fh; (k) the Fe 2p XPS spectra of ANF/PP‐Fh; (l) FTIR spectra of ANF/PP and ANF/PP‐Fh.

Biological membranes inspire approaches to overcome such transport limitations. In cell membranes, carrier proteins enable rapid ion transport across membranes (Figure 1b), achieving throughput far exceeding that of engineered membranes. Inspired by this principle, facilitated transport strategies based on specific carrier‐solute interactions have been investigated in engineered membranes, particularly for gas separation, metal ion recovery, and proton exchange membrane fuel cells [29, 30]. Recently, facilitated phosphate transport membranes have also attracted increasing attention, where phosphate‐binding carriers are incorporated to provide additional transport pathways beyond conventional diffusion or electromigration [31] (Figure 1c). Phosphate is a hard (Lewis) base and preferentially coordinates with hard Lewis acids such as ferric iron (Fe^3+^) [32], and the dominant mechanism of phosphate interacting with Fe oxides/hydroxides is inner‐sphere complexation associated with a ligand exchange process [15, 17]. Such chemical affinity has the potential to provide an additional driving force for fast mass transfer of phosphate [33]. Recent studies have demonstrated the effectiveness of facilitated phosphate transport membranes through the incorporation of manganese oxides into ion exchange membranes [34, 35, 36]. These studies have established the fundamental role of metal‐phosphate interactions in enhancing phosphate transport across membranes. Despite these important advances, translating facilitated phosphate transport concepts into high‐throughput e‐MC remains challenging. Existing studies have primarily focused on improving phosphate selectivity under diffusion‐driven or low‐flux separation conditions. Moreover, mechanistic insights into how phosphate‐carrier interactions and electric fields regulate ion transport behavior within membranes remain insufficiently understood. Therefore, developing electro‐driven membranes that integrate chemical affinity with electrochemical driving forces represents an important strategy for enhancing phosphate recovery performance.

Herein, we introduce an electro‐driven carrier‐conducting membrane (e‐CCM) designed to overcome the intrinsic kinetic limitations of phosphate transport. Our strategy leverages the synergistic interplay between electrochemical migration and targeted chemical affinity by integrating iron (oxy)hydroxide nanoparticles as phosphate carriers within the membrane matrix. To realize this, we develop a composite membrane featuring monodispersed ferrihydrite (Fh) nanoparticles assembled in situ within a polyamine cage and supported on Kevlar aramid nanofibers (ANFs). This unique carrier architecture provides abundant, spatially distributed coordination sites that function as a rapid phosphate transport network. Through synergistic chemical and electrochemical carriers, the resulting e‐CCMs enable fast phosphate extraction and high‐purity struvite production in an e‐MC system. This work extends facilitated transport concepts toward high‐throughput electro‐driven resource recovery applications and provides mechanistic insights into carrier‐conducting ion transport under electric fields.

## 2 Results and Discussion

### 2.1 Design and Characterization of e‐CCM

In this work, monodisperse Fh nanoparticles are designed as a phosphate carrier and hybridized within a polymeric matrix through their in situ formation in polyethyleneimine (PEI) and polyacrylonitrile (PAN), yielding a composite denoted as PP‐Fh (Figure S3). Visually, the PP‐Fh layer exhibits a brown color, whereas the carrier‐free polymer layer (PP) appears light yellow (Figure S4). To enhance mechanical robustness and antifouling performance, both PP‐Fh and PP layers are cast onto ANFs [37], resulting in the e‐CCM (ANF/PP‐Fh) and electro‐driven noncarrier membrane (ANF/PP), respectively (Figures S5, S6, and Table S1). Scanning electron microscopy (SEM) reveals a distinct difference in surface morphology between these resulting composite membranes. The ANF/PP membrane displays a fibrous and uneven surface, with valleys and depressions seen from the high‐magnification inset (Figure 1d). In contrast, the ANF/PP‐Fh exhibits a smoother and denser surface (Figure 1e). This morphological change is attributed to the incorporation of nanoscale Fe‐based carriers, which fill the voids in the fibrous structure. Cross‐sectional SEM images further confirm that the PP–Fh interlayer forms a compact and well‐integrated structure (Figure 1f), whereas the PP interlayer without carriers exhibits a more layered morphology (Figure S7). The filling of pores by Fe oxide nanoparticles thus leads to a denser membrane architecture [38, 39]. Elemental mapping by energy‐dispersive x‐ray spectroscopy (EDX) confirms a homogeneous Fe distribution throughout the membrane surface (Figure S8). However, individual Fe oxide particles are not distinguishable from the membrane matrix in the SEM images, possibly due to their fine dispersion and nanoscale dimensions. Transmission electron microscopy (TEM) of the ANF/PP–Fh membrane further reveals a uniform and compact carrier‐polymer structure without detectable aggregation (Figure 1g), indicating homogeneous nanoscale features within the PP–Fh layer. Consistently, the TEM–EDX mapping shows (Figure S9) that Fe is spatially distributed within the membrane matrix rather than being localized in a single discrete layer. This observation supports that the Fe‐based carrier is not limited to the central active layer but is integrated with the ANF layers, supporting the formation of a continuous membrane structure. High‐resolution TEM (HRTEM) identifies locally ordered lattice fringes indexed to the (110) plane (d‐spacing = 0.22 nm, Figure 1h), and the corresponding selected area electron diffraction (SAED) patterns indicate diffraction rings of 0.25, 0.22, 0.15, and 0.13 nm, suggesting 6‐Line Fh [40, 41]. Bulk x‐ray diffraction (XRD) measurements further confirm carrier formation in the ANF/PP‐Fh membrane (Figure 1i), showing two broad peaks at 2θ = 36° and 62°, corresponding to (110) and (115) planes of 2‐line Fh (Figure S10) [42]. These findings suggest that the dominant carrier (i.e., Fe oxide phase) in ANF/PP‐Fh is highly amorphous 2‐line Fh, while a minor fraction of 6‐line Fh is detected locally by HRTEM (Figure 1h). These Fh characteristic peaks are absent in the ANF/PP membrane (Figure 1i). X‐Ray photoelectron spectroscopy (XPS) analysis shows that the Fe content in the ANF/PP‐Fh membrane is approximately 3.5 wt% (Figure 1j). High‐resolution Fe 2p spectra display characteristic peaks at 711.2 and 724.7 eV, along with satellite features, confirming the presence of Fe(III) (Figure 1k) [43]. Additional information from high‐resolution O 1s XPS and Fourier‐transform infrared spectroscopy (FTIR) indicates Fe‐O bonding typical of Fe oxides (Figures 1l and S11) [44].

To further enhance ion mass transfer under an applied electric field, membranes rich in amino (−NH_2_) groups are functionalized with 2,3‐epoxypropyl trimethyl ammonium chloride (ETAC) monomer, using tris(dimethylaminomethyl)phenol (DMP‐30) as a catalyst to promote the epoxy groups of ETAC reacting with ‐NH_2_ groups in ANF/PP and ANF/PP‐Fh (Figure S12). The resulting quaternized membranes are denoted as QANF/PP and QANF/PP‐Fh. This functionalization introduces positively charged quaternary ammonium groups on the membrane surface, thereby strengthening the electrostatic attraction toward negatively charged phosphate and promoting their transport across the membrane. Compared with their non‐quaternized membranes (ANF/PP and ANF/PP‐Fh membranes, Figure 1d–f), SEM images of QANF/PP and QANF/PP‐Fh reveal rougher and more fibrous surface morphologies (Figures S7 and S8). XPS spectra further show increased O 1s and N 1s intensities after functionalization (Figure S13). The emergence of new C─N^+^ (285.8 eV)/N^+^‐C (402.1 eV) peaks, together with an enhanced C─O signal at 287.5 eV, confirms the successful grafting of quaternary ammonium groups via the reaction between ETAC and −NH_2_ groups (Figure S11) [22, 45].

Ion exchange membranes used for ion separation typically require a high ion exchange capacity to achieve efficient ion transport. Incorporation of Fh as a chemical phosphate carrier greatly increases the phosphate exchange capacity of the membrane (Figure 2a), rising from 1.43 mmol g^−1^ for ANF/PP to 2.09 mmol g^−1^ for ANF/PP‐Fh. This enhancement arises from the abundant surface hydroxyl groups (≡FeOH) on the carrier, which can undergo protonation to form ≡FeOH_2_^+^ and thereby function as additional anion‐exchange sites [46]. Importantly, the increased phosphate exchange capacity does not come at the expense of excessive membrane hydration or dimensional instability. As shown in Figure 2b, incorporation of the carrier leads to a decrease in water uptake from 27.9% to 21.8% and a reduction in swelling ratio from 6.4% to 5.7%. This behavior is attributed to the formation of a denser and more homogeneous internal carrier–polymer network, in which the confined Fh phase restricts polymer chain mobility and limits free volume available for water absorption [47]. Water contact angle measurements further demonstrate enhanced membrane hydrophilicity following carrier construction, evidenced by a lower water contact angle compared to the carrier‐free ANF/PP membrane (Figure 2c). This improvement in hydrophilicity can be attributed to two key factors: on the one hand, Fh itself is intrinsically hydrophilic, owing to its hydroxyl‐rich surface [45, 48]. On the other hand, the increased surface roughness of the ANF/PP‐Fh membrane relative to the ANF/PP membrane (Figure S14) may further amplify the hydrophilicity according to the Wenzel state of Young's model [49].

**FIGURE 2:**
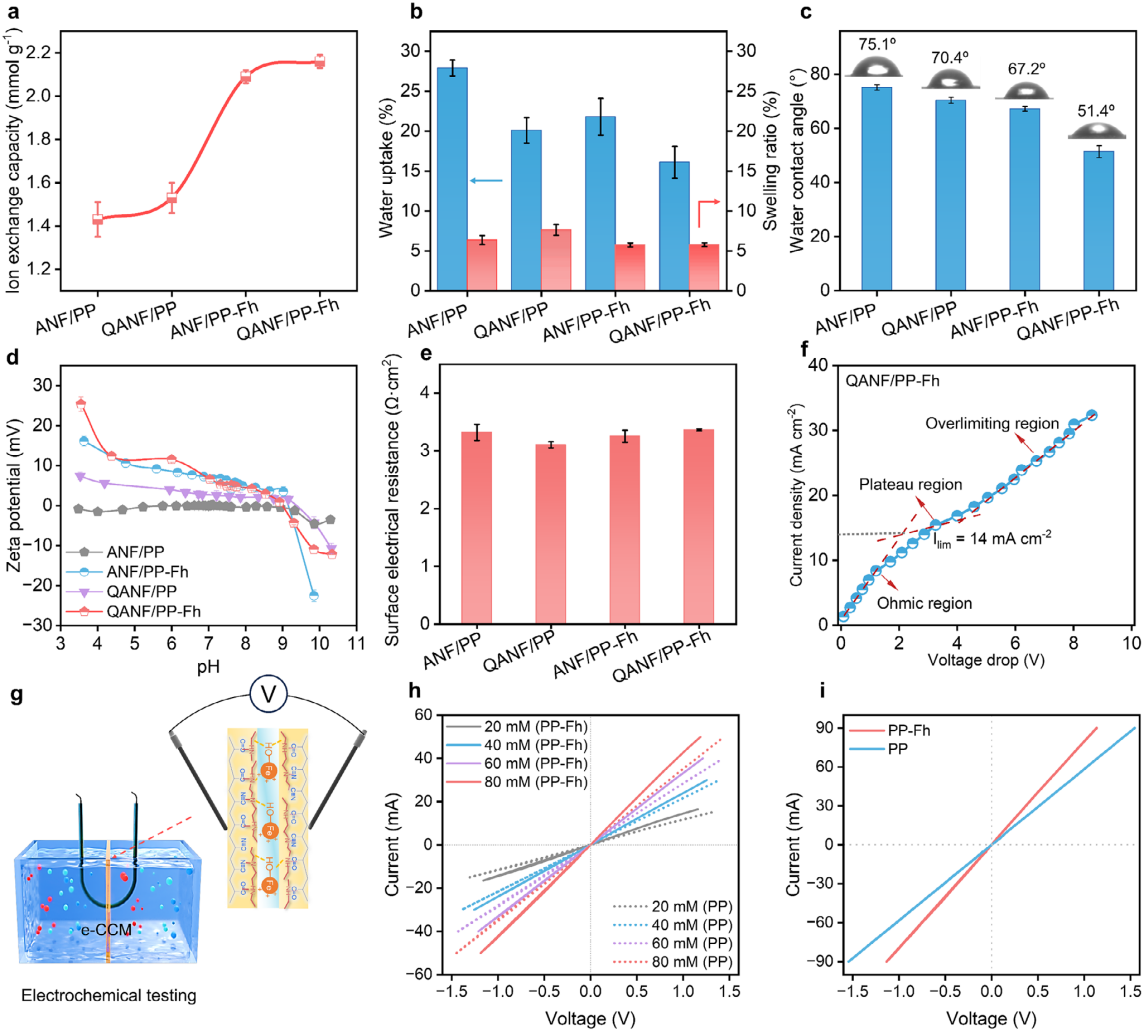
Membrane physicochemical and electrochemical properties. (a) Phosphate exchange capacities; (b) water uptake and swelling ratios; (c) water contact angle; (d) zeta potential of membranes; (e) surface electrical resistance of membranes measured in 0.5 M NaCl; (f) polarization C–V curves of the QANF/PP‐Fh membrane measured in 0.05 M NaCl solution; (g) schematic of the electro‐driven cell used for ion transport measurements; (h and i) I‐V curves of both e‐CCM and noncarrier membranes in unary NaH_2_PO_4_ solution and synthetic urine to elucidate the carrier‐conducting phosphate transport behavior. Error bars represent the standard error from triplicate measurements.

Zeta potential measurements further highlight the chemical impact of the carrier on membrane surface charge. While the ANF‐PP membrane exhibits a negatively charged surface, the ANF/PP‐Fh membrane maintains a positive surface charge across a broad pH range up to 9.0 (Figure 2d). This behavior is primarily governed by the intrinsic surface chemistry of Fh, which exhibits a relatively high zeta potential of approximately 20 mV at pH = 6 [50, 51]. The presence of positively charged ≡FeOH_2_^+^ sites thus also creates a favorable electrostatic environment for attracting anionic phosphate. The electrochemical behavior of the membrane is measured in a 0.5 M NaCl solution in a laboratory‐designed device (Figure S15). A lower surface electrical resistance is desirable, as it is directly associated with reduced energy consumption during electrodialysis operations [52]. Membranes with or without phosphate carrier exhibit comparable surface electrical resistances (Figure 2e), which are only slightly higher than that of the commercial AEM manufactured by FUJIFILM Europe BV (Japan) (i.e., referred to as AMX, Table S9) measured under the same conditions (2.7 Ω cm^2^). Consistently, all membranes display similar limiting current density (*I*_lim_) (Figures 2f and S16). Furthermore, Membranes functionalized with ETAC show enhanced surface properties. The phosphate exchange capacity of quaternized membranes (QANF/PP and QANF/PP‐Fh membranes) increases (Figure 2a), accompanied by a more positively charged surface (Figure 2d) and improved hydrophilicity (Figure 2c). These changes arise from the grafting of ETAC molecules containing quaternary ammonium groups via the substitution reaction between −NH_2_ (in ANF/PP) and the epoxy group in ETAC (Figure S12).

To directly probe carrier‐facilitated phosphate transport under an applied electric field, current‐voltage (I–V) measurements are conducted in both single phosphate solutions and simulated urine with mixtures of anions (i.e., Cl^−^, SO_4_^2−^, and H_2_PO_4_^−^) (Figure 2g). Across all NaH_2_PO_4_ concentrations, e‐CCM (i.e, PP‐Fh) exhibits steeper slopes in I–V curves compared to its non‐carrier counterpart (i.e., PP), indicating higher phosphate conductivity and faster charge transport (Figure 2h). Notably, this behavior persists in simulated urine (Figure 2i), demonstrating that Fe‐based carriers sustain rapid phosphate transport even in complex ionic environments.

### 2.2 Ion Separation Performance

Ion separation performance is evaluated using a laboratory‐scale four‐compartment electrodialysis cell with an effective area of 20 cm^2^ (Figure S17). At a current density of 5 mA cm^−2^ in a single‐phosphate solution, incorporation of Fh as carriers greatly enhances phosphate transport across the membrane. The phosphate concentration in the recovery chamber increases more rapidly for the ANF/PP–Fh membrane than for the carrier‐free ANF/PP membrane (Figure S18), resulting in a 1.45‐fold higher phosphate permeation rate after 100 min of electrodialysis (Figure 3a). Consequently, the phosphate recovery rate reaches 91.9% for the ANF/PP‐Fh membrane, compared to 82.9% for the ANF/PP membrane (Figure 3b).

**FIGURE 3:**
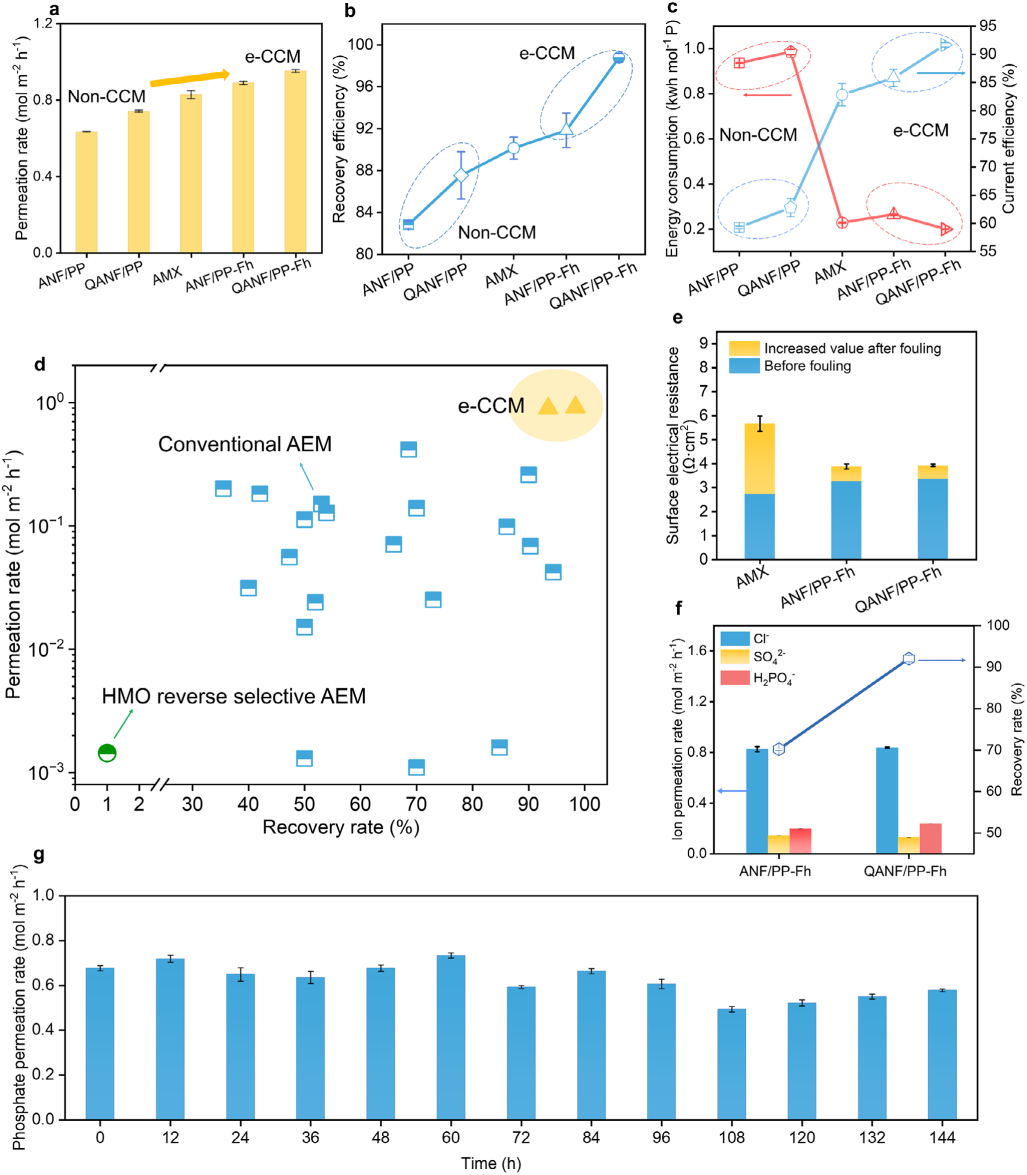
Membrane performance in phosphate recovery and antifouling ability. (a) Phosphate permeation rates of prepared membranes during 100 min of electrodialysis; (b) Phosphate recovery rates of membranes; (c) Energy consumption and current efficiency; Experimental conditions: the electrodialysis is run at a current density of 5.0 mA cm^−2^, the feed and concentrated solutions are initially composed of 100 mL 40 mM NaH_2_PO_4_ with pH = 6, 0.2 M Na_2_SO_4_ is used as electrode solution; (d) Comparison of phosphate permeation rate and recovery rate of membranes in electrodialysis; (e) the changes in surface electrical resistance after fouling; (f) ion permeation rate and phosphate recovery rate for the feed solution of synthetic urine: the electrodialysis is operated at a current density of 5.0 mA cm^−2^, the feed solution is synthetic fresh urine (Tables S4), the solution in the receiving chamber is initially composed of 50 mM NaCl; (g) Phosphate permeation rate of e‐CCM over 144 h. Error bars represent the standard error from duplicate measurements.

To decouple the contributions of electrostatic attraction from carrier‐mediated chemistry, membranes functionalized with quaternary ammonium groups (QANF/PP and QANF/PP‐Fh) are evaluated under identical conditions. Introduction of quaternary ammonium groups significantly enhances phosphate transport in the absence of carriers: the QANF/PP membrane exhibits a significantly higher phosphate permeation rate than ANF/PP (*p *< 0.01, Figure 3a), achieving a recovery of 87.6% (Figure 3b). This improvement is attributed to the positively charged quaternary ammonium groups, which facilitate the electromigration of negatively charged phosphate under the applied electric field. In contrast, quaternization produces only a marginal enhancement in phosphate transport for the carrier‐conducting membranes. The permeation rate of QANF/PP‐Fh increases slightly from 0.89 to 0.95 mol m^−2^ h^−1^ (*p *< 0.05, while the phosphate recovery reaches 98.8% (Figure 3a,b). This diminished sensitivity to additional fixed charges highlights a key mechanistic distinction: in ANF/PP‐Fh, phosphate transport is already dominated by carrier‐assisted coordination sites (≡FeOH_2_^+^). As a result, further electrostatic functionalization offers limited incremental benefit. All e‐CCMs, regardless of quaternization, outperform the commercial AMX membrane in both phosphate permeation rate and recovery (Figure 3b), underscoring the dominant role of Fe‐based carriers in enabling fast phosphate transport.

Energy consumption and current efficiency are further evaluated to assess the sustainability of the phosphate recovery process. As shown in Figure 3c, e‐CCMs consistently exhibit lower energy consumption (*p *< 0.001) and higher current efficiency (*p *< 0.001) than carrier‐free membranes. Among all tested membranes, the QANF/PP‐Fh membrane demonstrates the optimal balance, achieving a low energy consumption of 0.20 kWh mol^−1^ P along with a high current efficiency of 91.7%.

Figure 3d compares the phosphate permeation rate and recovery rate for the prepared membranes with those of previously reported ion‐exchange membranes used in electrodialysis (Table S3). These e‐CCMs exhibit both a significantly higher phosphate permeation rate and recovery, outperforming conventional AEMs as well as reserve‐selective hydrous manganese oxide (HMO)‐CEM. This performance enhancement underscores the critical role of carrier‐conducting transport introduced by the incorporation of Fh nanoparticles.

Beyond achieving high permeation rates, membrane fouling remains a critical challenge in membrane‐based separation processes, leading to reduced ion permeation, frequent cleaning, and a shorter operational lifespan [38, 53]. In this study, a mixed organic system consisting of urea and humic acid is employed to evaluate the antifouling performance of the e‐CCM. Urea represents the most abundant neutral organic species in urine, while humic acid is widely recognized as a model foulant for AEMs due to its negative charge, and its strong complexation ability with metal sites may form irreversible binding (i.e., membrane poisoning) [54, 55]. Although real urine contains more complex organic matter, including creatinine and uric acid, previous work on Fh has shown that small N‐containing metabolites (e.g., uracil) exhibit negligible interaction with Fe [56]. Therefore, the combination of urea and humic acid provides a representative yet conservative model system, capturing both dominant urine‐derived organics and strongly interacting foulants. Under these conditions, the e‐CCMs exhibit only a minor increase in surface electrical resistance after seven hours of electrodialysis (i.e., 0.57–0.63 Ω cm^2^, Figure 3e). In contrast, the hydrophobic commercial AMX membrane (with a water contact angle of 99.6°) experiences severe fouling, with resistance increasing by up to 2.94 Ω cm^2^. These results demonstrate that carrier‐conducting membranes simultaneously deliver high phosphate throughput and robust antifouling performance. Nevertheless, it should be noted that the current model system does not fully capture the compositional diversity of real urine. Further validation using real urine or more complex synthetic mixtures is therefore necessary to assess long‐term stability and the potential for irreversible occupation of active carrier sites.

To further validate carrier‐conducting phosphate transport in complex systems, electrodialysis tests are conducted using synthetic fresh urine containing competing anions and organic foulants (Table S2). ANF/PP‐Fh and QANF/PP‐Fh membranes, identified as optimal in terms of phosphate transport, energy efficiency, and antifouling performance, are selected for these experiments. The urine is freshly prepared before testing, and the composition is detailed in Table S4. Owing to its high concentration and small hydrated diameter, Cl^−^ dominates overall anion transport (Figure 3f and Table S5) [26]. Nevertheless, e‐CCMs exhibit preferential transport of H_2_PO_4_^−^ over SO_4_^2−^, even though SO_4_^2−^ typically shows higher mobility in conventional AEMs [57, 58]. This behavior contrasts with prior reports, such as that by McCartney et al., who observed higher SO_4_^2−^ permeation than H_2_PO_4_^−^ in polymeric AEMs despite lower SO_4_^2−^ concentrations (16 vs. 30 mM H_2_PO_4_^−^) [58]. Although Cl^−^ occurs at higher concentrations and exhibits greater absolute permeation, phosphate has a much stronger binding affinity for ≡FeOH sites. Consequently, Cl^−^ neither permanently occupies carrier sites nor hinders carrier‐to‐carrier hopping of phosphate. Moreover, the e‑MC prioritizes rapid, sustained phosphate transport to maximize productivity and enable efficient K‑struvite crystallization, whereas Cl^−^ does not crystallize. In mixed‐salt feeds, phosphate permeation decreases relative to single‐salt solutions due to lower phosphate concentration and competitive transport. Yet, the QANF/PP‐Fh membrane still achieves a high phosphate recovery of 92.1%.

Finally, the long‐term stability of the e‐CCM is evaluated using the QANF/PP‐Fh membrane under continuous electrodialysis operation for 144 h (Figure 3g). The membrane demonstrates a slowly decreasing phosphate permeation rate from 0.68 to 0.58 mol m^−2^ h^−1^. This slight decline is likely due to the partial occupation of active sorption sites during prolonged use. Nevertheless, the phosphate permeation rate is still significantly higher than that of conventional electro‐driven membranes, indicating the robustness of the e‐CCM. To further examine potential degradation of e‐CCMs, ICP‐OES analysis of the receiving solution is conducted throughout the stability test, and the Fe concentration remains below the quantification limit (Table S6), indicating negligible Fe leaching from the e‐CCM. In addition, XRD characterization of e‐CCM after operation shows no observable changes in the characteristic broad diffraction features of Fh and no detectable formation of secondary crystalline phases (Figure S19). These results further suggest that the Fh carriers remain chemically and structurally stable during prolonged operation.

### 2.3 Nonequilibrium Molecular Dynamics Simulations of Carrier‐Conducting Phosphate Transport

To elucidate the molecular origin of carrier‐conducting phosphate transport, non‐equilibrium molecular dynamics (MD) simulations are performed under an external electric field of 0.015 V Å^−1^ for the e‐CCM (i.e., PP‐Fh) and noncarrier membrane (i.e., PP) (Figures 4a and S20). In the PP–Fh system, Fe atoms, serving as chemically active phosphate carrier centers, are homogeneously dispersed throughout the polymeric matrix (Figures 4b and S21), ensuring spatial continuity of carrier sites across the membrane. Density profiles along the membrane's Z‐axis show that phosphate ions are initially located on the feed side and within the membrane interfacial region (Figure 4c). After 10 ns of simulation, phosphate ions are observed on the permeate side for both membranes (Figure 4d), demonstrating successful translocation of phosphate across the membrane under the applied electric field (Movies S1 and S2). The transport mechanisms are then further explored. It is important to note that the enhanced transport behavior in PP‐Fh cannot be attributed to differences in membrane pore structure. Structural analysis using Zeo++ shows that the pore size characteristics and porosity of PP and PP‐Fh membranes are nearly identical [59] (Table S7). In fact, the overall porosity slightly decreases in the PP‐Fh system. These results exclude the possibility that accelerated phosphate transport originates from enlarged transport channels or increased free volume in PP‐Fh. Therefore, the observed transport behavior is more reasonably attributed to specific phosphate‐carrier interactions. We hypothesize that phosphate transport in PP‐Fh follows a two‐step carrier‐conducting mechanism: (i) rapid carrier‐assisted diffusion at the water/membrane interface via phosphate‐Fe coordination (Figure S22), followed by (ii) electric‐field‐driven migration through successive carrier‐to‐carrier hopping events within the membrane. To validate this hypothesis, potential of mean force profiles (PMF) for phosphate permeation are calculated for both membranes. As shown in Figure 4e, phosphate possesses a larger PMF in the PP‐Fh membrane than in the PP membrane, indicating stronger phosphate‐carrier interactions. This is further supported by the sharper peak in the radial distribution function (RDF) (Figure 4f), reflecting a denser Fe‐O‐P coordination environment within the carrier network. Consistently, Importantly, the increased PMF does not impede phosphate transport. Instead, the strong yet reversible coordination between phosphate and ≡FeOH sites lowers the kinetic barrier for membrane entry and stabilizes transient carrier‐phosphate intermediates. In bulk solution, the average free diffusion energy of H_2_PO_4_^−^ is approximately 3.6 kcal mol^−1^. Under the applied electric field (0.015 V Å^−1^), an additional energetic contribution of 0.346 kcal mol^−1^ is available, yielding a total driving energy of 3.946 kcal mol^−1^. This value exceeds the maximum energy barrier observed in the PMF profile (i.e., 2.82 kcal mol^−1^), demonstrating that once the phosphate is captured by carrier sites and enters the membrane, it can readily overcome the energetic barriers and migrate directionally via carrier‐to‐carrier hopping. In addition, the linear relationship between conductance and temperature is obtained according to the Arrhenius formula for PP and PP‐Fh (Figure S23), which can be further calculated to obtain the activation energy (*E*_a_). On the one hand, a slightly higher *E*_a_ of PP‐Fh (2.83 kcal mol^−1^) than that of PP (2.60 kcal mol^−1^) is consistent with the stronger phosphate‐carrier interactions. On the other hand, the pre‐exponential factor (A) of PP‐Fh is significantly higher than that of PP, indicating an increased density of effective transport pathways and hopping events within the carrier network [60, 61]. Therefore, the combined MD simulations and experimental Arrhenius analysis consistently support a carrier‐conducting phosphate transport mechanism in the PP‐Fh membrane.

**FIGURE 4:**
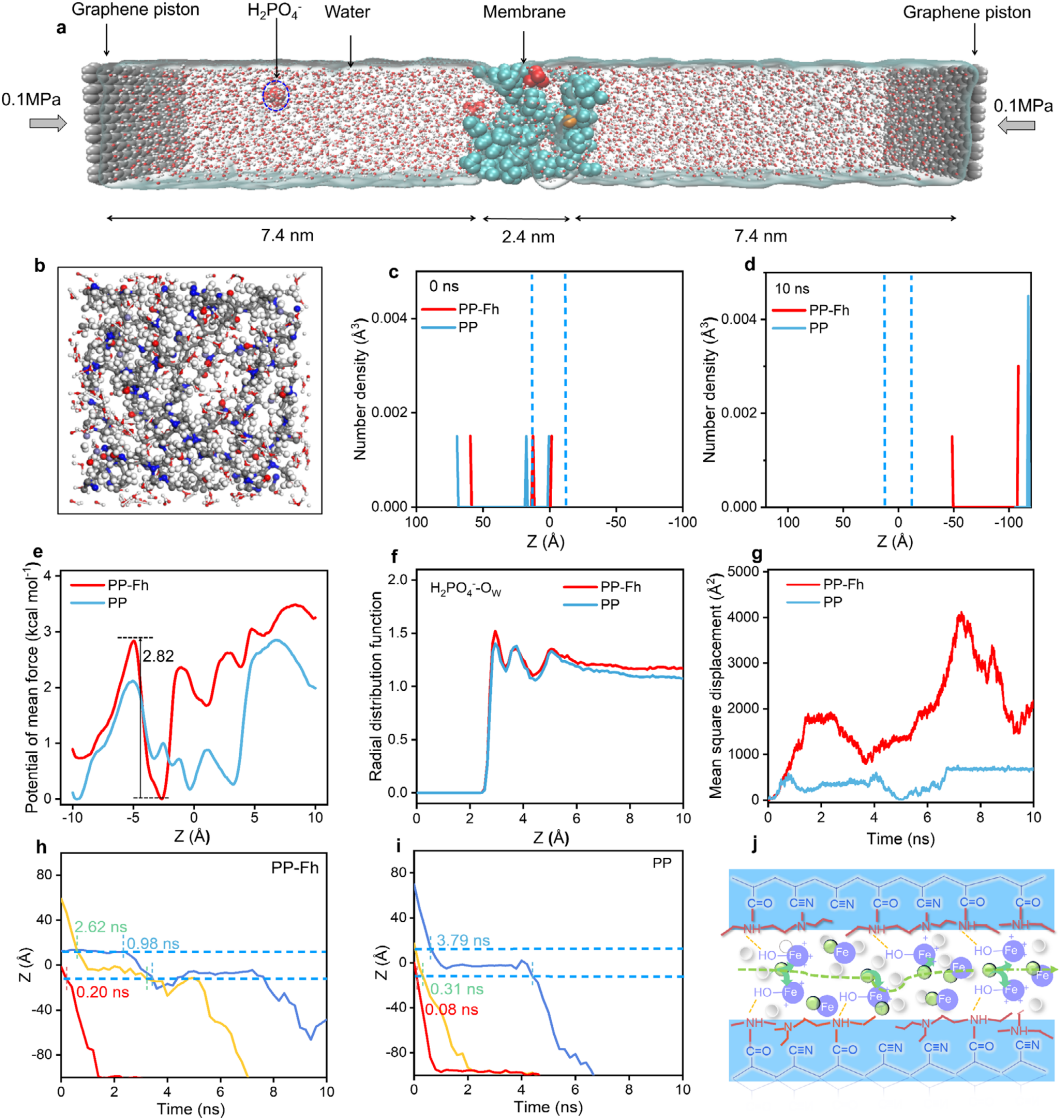
Molecular dynamics simulations of carrier‐conducting phosphate transport. (a) Set‐up of a non‐equilibrium model for phosphate transport through the PP‐Fh membrane. The PP‐Fh membrane with a thickness of 2.4 nm is positioned between two water chambers, with graphene pistons at two ends. 0.1 MPa is applied to both graphene pistons. The phosphate transport is driven by an electric field; (b) Computational model of the x‐y plane for the PP‐Fh membrane. The size of the cell is 24 × 24 Å. Color of atoms and ions: red‐oxygen, blue‐nitrogen, grey‐carbon, white‐hydrogen, purple‐Fe, the Fe atoms act as carrier centers for phosphate coordination and shuttling; (c, d) Z‐axis density distribution of phosphate at 0 and 10 ns, respectively. The area between two vertical lines represents the membrane; (e) Potential of mean force profiles for phosphate transport within membranes; (f) Radial distribution function of H_2_PO_4_^−^ and oxygen atoms in water molecules; (g) mean squared displacement of phosphate in the PP‐Fh membrane as a function of time; (h–i) Dynamic changes of three phosphate ions during the simulation time along Z‐axis in PP‐Fh and PP membranes. The area between two horizontal lines represents the membrane; (j) Schematic diagram of the carrier‐conducting phosphate transport pathway in e‐CCM, where phosphate hops between nanoscale Fe‐based carriers under an electric field, enabling fast permeation.

To further quantify phosphate mobility, mean square displacement (MSD) analyses are conducted. Phosphate exhibits a higher effective diffusivity in the PP‐Fh membrane than in the PP membrane (Figure 4g). Trajectory analysis of individual phosphate entering the membrane region along the Z direction reveals shorter residence times in the PP‐Fh membrane (2.62, 0.98, and 0.20 ns, with an average travel time of 1.27 ns) (Figure 4h), compared to the PP membrane (3.79, 0.31, and 0.08 ns, with an average of 1.39 ns) (Figure 4i). The reduced residence time corroborates the faster carrier‐conducting hopping in the PP‐Fh (Figure 4g). Combined with experimental evidence (Figure S24), the simulation results confirm that phosphate forms reversible inner‐sphere complexes with Fh, generating transient carrier‐phosphate intermediates that facilitate directional transport. Based on both experimental and computational data, we propose a carrier‐conducting hopping mechanism (Figure 4j), in which phosphate repeatedly coordinates to and dissociates from Fe(III) carrier sites within the Fh network under an electric field. This coordination‐decoordination relay transforms phosphate transport from a diffusion‐limited process into an energetically favorable, field‐biased hopping pathway, enabling rapid phosphate migration across the PP‐Fh membrane.

### 2.4 The e‐MC in a Lab‐Scale Electrodialysis Stack

To evaluate the practical applicability of e‐CCM, the QANF/PP‐Fh membrane is integrated into a lab‐scale e‐MC system for phosphate recovery and the production of high‐purity potassium‐struvite (K‐struvite, MgKPO_4_ˑ6H_2_O) from simulated urine (Figure 5a). As an initial membrane‐level demonstration toward practical implementation, the performance of the e‐CCM is further examined in an electrodialysis configuration with an enlarged membrane area (Figure 5b). For this purpose, the synthesized QANF/PP‐Fh is assembled into an electrodialysis stack with an effective area of 60 cm^2^ and benchmarked against a commercial AMX membrane (Table S9). The membrane set consists of one QANF/PP‐Fh membrane (Figure 5c), one AMX, and two CMXs (Figures S25 and S26), forming a multi‐compartment ED configuration for evaluating phosphate transport and its coupling with crystallization (Figure 5d,e). The e‐MC system is operated using synthetic urine (Table S2) under a constant voltage of 5 V (Figure S27).

**FIGURE 5:**
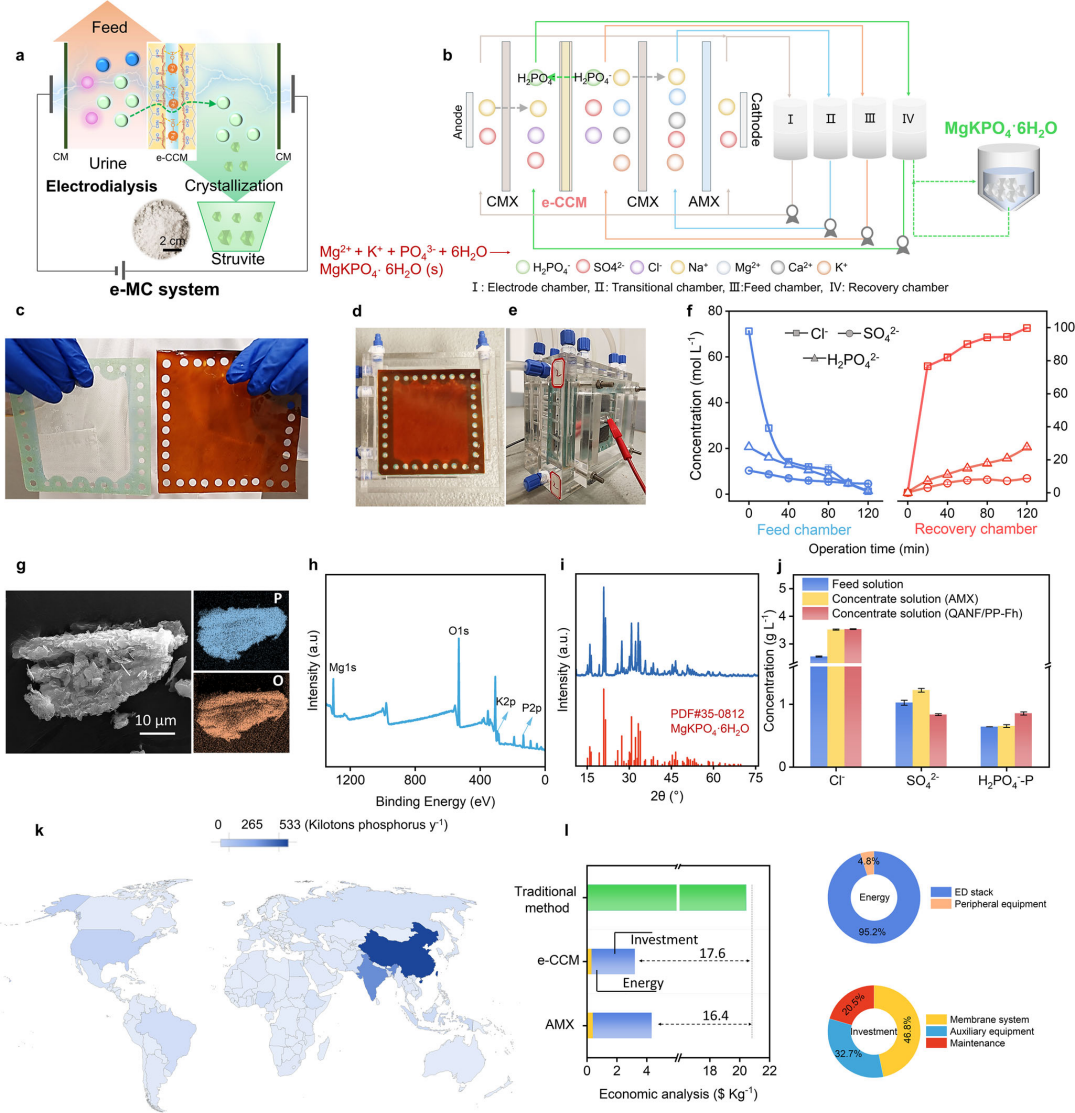
Phosphate extraction from urine and K‐struvite (MgKPO_4_ˑ6H_2_O) production in a laboratory‐scale electrodialysis stack operated at an optimized voltage of 5 V, using synthetic urine as the initial feed. (a) Schematic of e‐MC of K‐struvite, the photo shows K‐struvite powder; (b) the configuration of e‐MC in the electrodialysis stack for phosphate extraction and K‐struvite precipitation from urine; (c) photos of membrane and spacer; (d) photo of the membrane assembled in the electrodialysis stack; (e) Photo of one electrodialysis stack in operation; (f) temporal evolution of anions in feed and recovery chambers of the QANF/PP‐Fh membrane; (g) SEM images of K‐struvite crystals and element mapping of P and O distribution; (h) XPS survey spectra of the K‐struvite crystals; (i) XRD patterns of the precipitates, confirming the crystallographic phase of struvite (PDF#35‐0812); (j) compositions of initial feed solution and concentrate solution after testing of AMX and QANF/PP‐Fh membrane; (k) a rough estimate of the recovered phosphate yield from phosphate produced in urine each year by our e‐MC system across 189 countries; (l) Comparison in economic analysis of e‐CCM in e‐MC system with AMX and traditional struvite precipitation method. Error bars represent the standard error from duplicate measurements.

The anion concentration profiles in the feed and recovery chambers are shown in Figures 5f and S24. Consistent with results obtained in single‐membrane cells, the electrodialysis stack clearly demonstrates accelerated phosphate transport enabled by the carrier‐conducting membrane. After 120 min of operation, the H_2_PO_4_^−^ concentration in the feed chamber decreases to 1.4 mM, while it increases to 27.6 mM in the recovery chamber (Figure 5f). In contrast, the AMX reduces H_2_PO_4_^−^ concentration only to 6.4 mM in the feed chamber and increases it to 21.1 mM in the recovery chamber (Figures S28 and S29). The concentrated phosphate solution is subsequently reacted with MgCl_2_ and KCl to precipitate K‐struvite, a slow‐release fertilizer capable of delivering both P and K to plants [62]. SEM images of the recovered precipitate reveal irregular and flaky crystal structures, and elemental mapping confirms the homogeneous distribution of P, O, Mg, and K in the crystals (Figures 5g and S30). XPS pattern confirms the presence of Mg, P, O, and K (Figure 5h). The XRD analysis (Figure 5i) further confirms the crystalline phase of the product as pure MgKPO_4_ˑ6H_2_O (PDF#35‐0812), with no detectable impurities. Figure 5j summarizes the anion concentrations in the feed and recovery chambers. With the QANF/PP‐Fh membrane, phosphate is concentrated by a factor of 1.46, compared to only 1.07 for the AMX membrane, during 120 min of e‐MC operation. Moreover, the molar ratio of H_2_PO_4_^−^/SO_4_^2−^ in the recovery chamber increases from 2.0 (in the feed) to 3.2, indicating preferential transport of H_2_PO_4_^−^ over SO_4_^2^. In contrast, the AMX membrane exhibits a decrease in this ratio to 1.6, suggesting preferential transport of SO_4_^2−^ over H_2_PO_4_^−^. This enhanced phosphate permeation of the QANF/PP‐Fh membrane results in a high phosphate recovery rate of 93.6%, significantly outperforming the AMX membrane, which achieves only 61.6% recovery (Figure S31). To assess the broader implications of this technology, we estimate the theoretical global potential for phosphate recovery from human urine using the e‐MC system across 189 countries (Figure 5k and Data S1). For example, in China alone, this approach could recover approximately 533 kilotons of phosphate annually from urine, corresponding to ∼11% of national phosphate fertilizer demand. These projections illustrate the potential societal impact of carrier‐conducting e‐MC technology.

Beyond performance, the e‐MC system integrating e‐CCMs offers significant techno‐economic and environmental advantages. Recovering phosphate from source‐separated urine can alleviate environmental pollution caused by excess phosphate discharge while generating valuable fertilizers that support sustainable agricultural productivity. To evaluate the economic viability of the system, a cost‐benefit analysis of the e‐MC is calculated based on the work of Yang et al. [63] (Figure 5l and Table S8). The total process costs are mainly categorized into energy and investment costs. The e‐MC system yields a total cost of 3.21 $ kg^−1^ K‐struvite, consisting of 0.31 $ kg^−1^ K‐struvite for energy and 2.90 $ kg^−1^ K‐struvite for investment. This expenditure is 1.13 $ kg^−1^ K‐struvite lower than the cost of operating the system with conventional AMX. Combined with the e‐CCM, the e‐MC can save up to 17.6 $ kg^−1^ K‐struvite in cost compared to the high cost of the traditional urine treatment method (i.e., 20.44 $ kg^−1^ K‐struvite) [63], highlighting its compelling techno‐economic benefits.

## 3 Conclusion

By leveraging principles of synergistic chemical and electrochemical carriers, this work designs an electro‐driven carrier‐conducting membrane (e‐CCM) that enables fast phosphate transport in electro‐driven membrane crystallization for efficient and sustainable phosphorus recovery. Experimental investigations and molecular dynamics simulations reveal that the accelerated phosphate permeation of the e‐CCM originates from carrier‐conducting transport, where Fe‐based carriers of ferrihydrite nanoparticles form transient Fe‐O‐P complexes that promote phosphate uptake and directional migration under an applied electric field. The e‐CCM demonstrates fast phosphate permeation and high phosphate recoveries in both single phosphate solutions and simulated urine, producing high‐purity struvite fertilizers. Moreover, the fabrication of these membranes shows potential for scale‐up and integration into large membrane stacks. Future research should focus on tuning carrier chemistry to enhance phosphate selectivity over competing anions, thereby further optimizing recovery performance. The carrier‐conducting design concept, technological principle, and evaluation methods established in this work guide the development of high‐throughput electro‐driven membranes framed for e‐MC systems.

## Author Contributions

**Lei Xia**: conceptualization, methodology, software, data curation, investigation, validation, formal analysis, resources, writing – original draft, writing – review & editing, visualization. **Yangbo Qiu**: methodology, data curation, investigation, formal analysis, visualization, writing – review & editing. **Qingzhi Liu**: software, formal analysis, data curation. **Mengjiao Guan**: software, data curation, formal analysis. **Xi Zhang**: writing – review & editing, data curation. **Raf Dewil**: writing – review & editing, formal analysis, investigation. **Jin Shang**: writing – review & editing, formal analysis. **Yan Zhao**: conceptualization, methodology, data curation, investigation, validation, formal analysis, funding acquisition, writing – review & editing, project administration, resources, supervision. **Bart Van der Bruggen**: conceptualization, writing – review & editing, formal analysis, resources. **Chuyang Tang**: conceptualization, writing – review & editing, formal analysis, resources.

## Conflicts of Interest

The authors declare no conflicts of interest.

## Supporting information




**Supporting File 1**: anie74486‐sup‐0001‐SuppMat.docx.

**Supporting File 2**: anie74486‐sup‐0002‐S1.mp4.

**Supporting File 3**: anie74486‐sup‐0003‐S2.mp4.

## Data Availability

The data that support the findings of this study are available from the corresponding author upon reasonable request.
