# Supplementary material for: Accelerating Phosphate Transport Across Membranes via Synergistic Chemical‐Electrochemical Carriers
Source: Angew Chem Int Ed Engl. 2026 Aug 27;65(43):e4185923. doi: 10.1002/anie.4185923 (PMC13676418; doi:10.1002/anie.4185923)
Supplement: Supplementary file 1 [file ANIE-65-e4185923-s002.docx]

**Supplementary Information for**

**Accelerating Phosphate Transport Across Membranes via Synergistic Chemical-Electrochemical Carriers**

Lei Xia ^1, 2, ||^, Yangbo Qiu ^1, ||^, Qingzhi Liu ^1, 3, ||^, Mengjiao Guan ^4^, Xi Zhang ^5^, Raf Dewil ^6, 7^, Jin Shang ^8^, Yan Zhao ^1,^ *, Bart Van der Bruggen ^9^, Chuyang Tang ^1^

^1^ Department of Civil Engineering, The University of Hong Kong, Pokfulam, SAR 999077, Hong Kong.

^2^ Division of Soil and Water Management, KU Leuven, Kasteelpark Arenberg 20, B-3001 Leuven, Belgium.

^3^ College of Chemistry and Pharmaceutical Sciences, Qingdao Agricultural University, Qingdao 266109, China.

^4^ College of Chemistry and Chemical Engineering, Ocean University of China, 266100, Qingdao, China.

^5^ Pritzker School of Molecular Engineering, University of Chicago, Chicago, Illinois 60637, United States.

^6^ Department of Chemical Engineering, KU Leuven, Process and Environmental Technology Lab, J. De Nayerlaan 5, 2860 Sint-Katelijne-Waver, Belgium.

^7^ Department of Engineering Science, University of Oxford, Parks Road, Oxford OX1 3PJ, United Kingdom.

^8^ School of Energy and Environment, City University of Hong Kong, Tat Chee Avenue, Kowloon, Hong Kong.

^9^ Department of Chemical Engineering, KU Leuven, Celestijnenlaan 200F, B-3001 Leuven, Belgium.

**^||^** These authors contributed equally to this work.

*Corresponding authors: [yanzhaox@hku.hk](mailto:yanzhaox@hku.hk) (Y. Zhao)

Table of Contents

[Experimental Section	5](#_Toc233753707)

[Figure S1. Schematic illustration of the global phosphorus cycle and electro-driven phosphate recovery.	12](#_Toc233753708)

[Figure S2. Global distribution of phosphate rock reserves (million tonnes P_2_O_5_) and annual phosphate consumption (kilotons P_2_O_5_ yr^-1^) (a), Long-term average total P (TP) concentrations in surface waters over the period 2000−2022 (b).	13](#_Toc233753709)

[Figure S3. Mechanism of the chemical reaction in the PP-Fh structure.	14](#_Toc233753710)

[Figure S4. Photographs of PP (left) and PP-Fh (right).	15](#_Toc233753711)

[Figure S5. The process of preparation of the Kevlar hydrogel membrane substrate (ANF).	16](#_Toc233753712)

[Figure S6. Schematic diagram of the structure of e-CCM, including the PP-Fh layer and ANF substrate.	17](#_Toc233753713)

[Figure S7. The cross-section SEM images of ANF/PP (a), QANF/PP (b), ANF/PP-Fh (c), and QANF/PP-Fh (d).	18](#_Toc233753714)

[Figure S8. The surface, zoomed-in surface, and EDX mapping images of ANF/PP (a1-a3), QANF/PP (b1-b3), ANF/PP-Fh (c1-c3), and QANF/PP-Fh (d1-d3).	19](#_Toc233753715)

[Figure S9. Cross-sectional TEM and EDX mapping images of ANF/PP-Fh.	20](#_Toc233753716)

[Figure S10. XRD patterns of PP-Fh and the synthesized 2-line ferrihydrite.	21](#_Toc233753717)

[Figure S11. XPS spectra of C 1s, N 1s, and O 1s high-resolution of ANF/PP (a1-a3), QANF/PP (b1-b3), ANF/PP-Fh (c1-c3), and QANF/PP-Fh (d1-d3).	22](#_Toc233753718)

[Figure S12. Mechanism of the chemical reaction in the QANF/PP (QANF/PP-Fh) structure.	23](#_Toc233753719)

[Figure S13. XPS spectra of the surface elements (atomic%) of QANF/PP and QANF/PP-Fh.	24](#_Toc233753720)

[Figure S14. The AFM height mapping with a scanning window of 5 μm × 5 μm of ANF/PP, ANF/PP-Fh, QANF/PP, and QANF/PP-Fh.	25](#_Toc233753721)

[Figure S15. Schematic of the electro-driven cell used for measurements of electrochemical properties.	26](#_Toc233753722)

[Figure S16. Polarization current density-voltage (C-V) curves of the AMX, ANF/PP, QANF/PP, and ANF/PP-Fh measured in 0.05 NaCl.	27](#_Toc233753723)

[Figure S17. Schematic of ion separation in electrodialysis.	28](#_Toc233753724)

[Figure S18. Phosphate concentrations in the recovery chamber. Error bars represent the standard error from triplicate measurements.	29](#_Toc233753725)

[Figure S19. XRD patterns of e-CCM after 144 hours of electrodialysis operation.	30](#_Toc233753726)

[Figure S20. Snapshots of non-equilibrium models of phosphate transport through e-CCM and non-carrier membrane at different states.	31](#_Toc233753727)

[Figure S21. Computational model of the x-z plane for PP-Fh membrane.	32](#_Toc233753728)

[Figure S22. Schematic illustration of the carrier-mediated mechanism for phosphate transport.	33](#_Toc233753729)

[Figure S23. Arrhenius plot of 40 mM NaH_2_PO_4_ conductance through PP and PP-Fh.	34](#_Toc233753730)

[Figure S24. XPS spectra for QANF/PP and QANF/PP-Fh after electrodialysis with 40 mM P solution.	35](#_Toc233753731)

[Figure S25. Photos of AMX (left) and CEM (right).	37](#_Toc233753732)

[Figure S26. Photo of the membrane assembled in the electrodialysis stack.	38](#_Toc233753733)

[Figure S27. Polarization current density-voltage (C-V) curves of the QANF/PP-Fh measured in simulated urine.	39](#_Toc233753734)

[Figure S28. Temporal evolution of anions in the feed and recovery chambers of AMX under an optimized voltage of 5 V.	40](#_Toc233753735)

[Figure S29. Temporal evolution of phosphate in the feed and recovery chambers of AMX and QANF/PP-Fh. Error bars represent the standard error from duplicate measurements.	41](#_Toc233753736)

[Figure S30. SEM images of K-struvite and element mapping of Mg and K distribution.	42](#_Toc233753737)

[Figure S31. Comparison in phosphate recovery rate of AMX and QANF/PP-Fh membranes in e-MC.	43](#_Toc233753738)

[Supplementary Tables	44](#_Toc233753739)

[Table S1. Names of the prepared membranes.	44](#_Toc233753740)

[Table S2. The recipe for preparing a fresh urine solution.	45](#_Toc233753741)

[Table S3. Comparison of the phosphate separation performance of electro-driven membranes in the electrodialysis system in the literature.	46](#_Toc233753742)

[Table S4. The measured ion concentration in the prepared fresh urine.	47](#_Toc233753743)

[Table S5. The hydrated diameters of anions [24]	48](#_Toc233753744)

[Table S6. Fe concentrations in the recovery chamber over 144 hours of electrodialysis operation.	49](#_Toc233753745)

[Table S7. Pore structure parameters and porosity of PP and PP-Fh membranes based on Zeo++ analysis [25].	50](#_Toc233753746)

[Table S8. The economic analysis of phosphate recovery	51](#_Toc233753747)

[Table S9. Characteristics of the FUJIFILM ion exchange membranes	53](#_Toc233753748)

[Movie S1.	54](#_Toc233753749)

[Molecular dynamics trajectory animation of e-CCM.	54](#_Toc233753750)

[Movie S2.	55](#_Toc233753751)

[Molecular dynamics trajectory animation of non-carrier membrane.	55](#_Toc233753752)

[Data S1.	56](#_Toc233753753)

[Average national total protein and vegetable protein consumption data and global phosphate fertilizer consumption data.	56](#_Toc233753754)

[Data S2.	57](#_Toc233753755)

[Global distribution of phosphate rock reserves, annual phosphate consumption, and long-term average total P concentrations in surface waters.	57](#_Toc233753756)

[Data S3.	58](#_Toc233753757)

[The economic analysis of phosphate recovery.	58](#_Toc233753758)

[References	59](#_Toc233753759)

Experimental Section

**Materials**

Kevlar nanofibers (K29) were obtained from DuPont (The United States). Polyacrylonitrile (PAN, average Mw = 150000), polyethyleneimine (PEI, average Mw = 25000), Dimethyl sulfoxide (DMSO), 2,3-epoxypropyl trimethyl ammonium chloride (ETAC), tris(dimethylaminomethyl)phenol (DMP30), potassium hydroxide (KOH, ≥ 99.95%), sodium phosphate monobasic dihydrate (NaH_2_PO_4_·2H_2_O), sodium sulfate (Na_2_SO_4_, anhydrous, 99%), sodium chloride (NaCl, 99.5%), magnesium chloride hexahydrate (MgCl_2_ˑ6H_2_O, 99%), calcium chloride dihydrate (CaCl_2_ˑ2H_2_O, 99%), potassium chloride (KCl, 99.9%), sodium hydroxide (NaOH, 99%), urea (molecular biology, ≥ 98%), acrylamide solution (40% w/w, Electron, BDH) and humic acid were purchased from Sigma Aldrich. Iron(III) chloride (FeCl_3_, anhydrous, 98%) was obtained from Thermo Scientific. Iron nitrate nonahydrate (Fe(NO_3_)_3_ˑ9H_2_O) and potassium hydroxide (KOH, 99%) were purchased from Fisher Chemical.

All the chemicals were used without further purification. The commercial AEM (Type I) and CEM (Type I) were purchased from FUJIFILM Manufacturing Europe BV (Japan), and the characteristics are shown in Table S9.

**Preparation of Kevlar aramid nanofibers casting solution**

Kevlar aramid nanofibers (ANF), synthesized from poly(p-phenylene terephthalamide) (PPTA), represent a class of advanced materials known for their remarkable mechanical strength and thermal stability. The ANF casting solution was prepared by dispersing ANFs in a DMSO solution containing KOH, with an ANF mass fraction of 2.5%. The mixture was magnetically stirred at room temperature until a homogeneous and stable ANF dispersion was obtained. During this process, hydrogen bonds, π-π stacking interactions, and van der Waals forces between the aramid nanofibers were broken, and the long amide chain was split into smaller segments. The ANF casting solution was sonicated before use to remove air bubbles.

**Preparation of PP-Fe^3+^ casting solution**

The PAN was dissolved in DMSO at a mass fraction of 7.5% under magnetic stirring at room temperature until a clear and homogeneous solution was obtained. The PEI was then added to the PAN solution and stirred continuously for 12 h at room temperature, yielding a uniform PAN/PEI (PP) casting solution with a PAN/PEI mass ratio of 2:1. The PP-Fe³⁺ casting solution, FeCl₃ was subsequently introduced into the PAN/PEI solution and stirred at room temperature until complete complexation of Fe³⁺ with PEI and a homogeneous PP-Fe³⁺ casting solution was formed. Before use, all casting solutions were bath-sonicated for 1 h to remove entrapped air bubbles.

**Membrane synthesis**

The e-CCMs were fabricated using a sequential synthesis method. First, the defoamed 2.5% ANF casting solution was slowly poured onto a clean glass plate and uniformly spread using a casting knife with a gap height of 100 μm at a constant speed. The resulting ANF hydrogel layer was dried in an oven at 60 °C to form an ANF support layer. Subsequently, the PP-Fe^3+^ casting solution was evenly coated onto the dried ANF layer using a casting thickness of 500 μm, followed by drying in the oven at 60 °C. During this step, Fe³⁺ ions complexed with PEI and were converted into ferrihydrite (Fh) upon subsequent hydration, yielding an intermediate membrane denoted as PP–Fh. A second ANF layer was then cast onto the PP-Fh layer with a thickness of 250 μm. After solvent evaporation, the glass plate was immersed in DI water, allowing the composite membrane to detach naturally. The resulting ANF/PP-Fh membrane exhibited a total thickness of approximately 46 µm, as measured by cross-sectional SEM. For comparison, ANF/PP membranes were prepared using a PAN/PEI casting solution without FeCl_3_, following the same fabrication procedure described above. The resulting ANF/PP membrane exhibited a comparable total thickness of approximately 44 µm.

The resulting composite membranes (both ANF/PP-Fh and ANF/PP) with dimensions of 8 cm × 25 cm × 850 μm were subsequently immersed in 1 L of an aqueous solution containing 10 g L⁻¹ ETAC. DMP-30 was added as a catalyst to initiate the quaternization reaction. After completion, the membranes were thoroughly rinsed with DI water to remove residual reagents and stored in DI water before use. The quaternized membranes were denoted as QANF/PP-Fh and QANF/PP, respectively.

**Synthesis of 2-line ferrihydrite in bulk**

The 2-line ferrihydrite was synthesized according to a typical method [1]. 40.0 g of Fe(NO_3_)_3_ˑ9H_2_O was dissolved in 500 mL ultrapure water, followed by the addition of 1.0 M KOH to bring the solution pH to 7-8 under vigorous stirring. Afterward, the precipitates were centrifuged and washed until free from electrolytes. The sample was freeze-dried before XRD characterization.

**Membrane Characterization**

**Water uptake, swelling ratio, and ion exchange capacity.** The water uptake (%) of AEM is determined by the weight difference of wet and dry AEM according to equation (1). The swelling ratio (%) is determined by the length difference of wet and dry AEM according to equation (2). The length and weight of the dry membrane were obtained ($\text{M}_{\text{dry}}$ and $\text{L}_{\text{dry}}$) after drying at 60 °C for 12 h. After this, the AEM was immersed in pure water and shaken for 24 h, then the water attached to the AEM was removed by tissue, and the length and weight of the wet AEM ($\text{M}_{\text{wet}}$ and $\text{L}_{\text{wet}}$) were measured.

$\text{WC = }\frac{\text{M}_{\text{wet}}\text{-}\text{M}_{\text{dry}}}{\text{M}_{\text{dry}}}\text{×100\%}$	(1)

$\text{SW = }\frac{\text{L}_{\text{wet}}\text{-}\text{L}_{\text{dry}}}{\text{L}_{\text{dry}}}\text{×100\%}$	(2)

The ion exchange capacity (IEC, mmol g^-1^) of AEMs is measured by using a titration method [2]. The AEM was first immersed in 50 mL 0.5 M NaCl for 48 h, then the AEM was rinsed with pure water and immersed in 50 mL 0.5 M NaNO_3_ for another 48 h, during which the Cl^-^ exchanges with NO_3_^-^ and is released to the solution. The Cl^-^ was measured by titration with AgNO_3_ (0.01 M) with K_2_CrO_4_ as an indicator. The ion exchange capacity is calculated according to equation (3).

$\text{IEC = }\frac{\text{(}\text{V}_{\text{s}}\text{-}\text{V}_{\text{b}}\text{)×}\text{C}_{\text{Ag}\text{NO}_{\text{3}}}}{\text{M}_{\text{dry}}}\text{×100\%}$	(3)

where $V_{s}$ is the volume of consumed AgNO_3_ solution with AEM, $V_{b}$ is the volume of consumed AgNO_3_ solution without AEM. $M_{dry}$ is the weight of the dry membranes (g).

**Attenuated total reflection Fourier transforms infrared (ATR-FTIR).** The functional groups of prepared AEMs were characterized by Fourier transform infrared spectroscopy (FTIR) with an attenuated total reflection (ATR) diamond accessory (FTIR-ATR, Brukerruker Alpha II, Germany).

**X-ray photoelectron spectroscopy (XPS).** The surface elemental compositions of the prepared membranes and K-struvite powders were measured by an X-ray photoelectron spectrometer (XPS, AXIS UltraDLD, Japan).

**Scanning electron microscopy (SEM).** The surface morphologies of AEMs and K-struvite powders were observed using a scanning electron microscope (SEM, JSM-7800 F, Japan), and element distribution mapping was collected on an energy-dispersive X-ray spectroscopy (EDS, INCA X-Act) analyzer.

**Transmission electron microscopy (TEM).** The TEM images were obtained using Tecnai G2 F20 (Thermo Fisher Scientific) at an accelerating voltage of 200 kV. The instrument provided a point resolution of ≤0.24 nm, a lattice (high-resolution) resolution of ≤0.102 nm, and an information resolution of ≤0.14 nm.

**Atomic force microscopy (AFM).** The three-dimensional topography and roughness of the AEMs surfaces were obtained by atomic force microscope (AFM, Nanonavi E-Sweep, Japan).

**Powder X-ray diffraction (XRD).** The crystallographic characterization of ANF/PP, ANF/PP-Fh, 2-line ferrihydrite, and K-struvite powders was identified by X-ray diffractometry (XRD-6100, Shimadzu, Japan) in the 2θ range of 10-80° at room temperature.

**Zeta potential.** The surface charge of prepared AEMs was analyzed using a zeta potential analyzer (EKA, SurPASS 3, Anton Paar) in a 1.0 mM KCl background solution with a pH range of 3.0–10.0.

**Water contact angle.** Water contact angle was measured on a contact angle goniometer (Dropmeter A-200, MAIST).

**Membrane electrochemical properties**

**Surface electrical resistance.** Surface electrical resistance (R) of all prepared AEMs and AMX was measured in a 0.5 M NaCl solution. The 0.2 M Na_2_SO_4_ was used as an electrode solution. Before testing, all membranes had been immersed in a 0.5 M NaCl solution for 8 h. Membrane surface electrical resistance R (Ω·cm^2^) is calculated as

$\text{R}_{\text{S}}\text{ = }\frac{\text{U-}\text{U}_{\text{0}}}{\text{I}}\text{×S}$	(4)

where U is the measured voltage when AEM is assembled in the device, U_0_ (V) is the voltage without AEM, I is the constant current through the membranes (0.04 A), and S is the membrane effective area (7.065 cm^2^).

**Polarization C-V curves**. Polarization C-V curves of the membranes were tested in 0.05 M NaCl in electrodialysis. Before testing, the membranes were immersed in a 0.05 M NaCl solution for 8 h. Membrane surface current density, I_s_ (A·cm^-2^), is calculated by

$\text{I}_{\text{s}}\text{ = }\frac{\text{I}}{\text{s}}$	(5)

where I (A) is the current through the membranes and S is the membrane effective area (7.065 cm^2^).

**I-V curves.** The dynamic behavior of membrane ion conductivities is evaluated through the I-V characteristics in 20 mM, 40 mM, 60 mM, 80 mM NaH_2_PO_4,_ and synthetic urine, respectively, in the laboratory-designed testing device. An electrochemical workstation (Autolab PGSTAT 302 N, Metrohm Belgium) is used to measure the PP-Fh and PP (7.065 cm^2^), and the scale of voltage is -1.5 V to 1.5 V.

**Antifouling measurements.** Antifouling performance of the membranes was measured in electrodialysis using a mixture solution of 20 g L^-1^ urea, 0.1 g L^-1^ humic acid, and 0.1 M NaCl. The membrane surface electrical resistance was measured before and after seven hours of electrodialysis under a membrane current density of 5 mA cm^-2^. The increase in the membrane surface electrical resistance was used to reflect the membrane's resistance to fouling.

**Electrodialysis separation tests**

This study uses two kinds of lab-scale electrodialysis stacks. For the membrane property analysis, a custom-designed device will be used, with the effective area of each membrane being 20 cm^2^. For the resulting membranes application for phosphate recovery in e-MC, the effective area of each membrane is 60 cm^2^.

The phosphate separation performances of the prepared membranes are evaluated in a custom-designed device, using single phosphate solutions. First, 100 mL single phosphate solution (40 mM, pH = 6) is used as feed and concentrate in electrodialysis at an applied current of 0.1 A (i.e., 5 mA cm^-2^, membrane current density) to compare the phosphate transport ability between e-CCM and non-carrier membrane. The 0.2 M Na_2_SO_4_ is used as an electrode solution. A commercial AEM (AMX) is applied in electrodialysis under the same conditions to compare the membrane performance. The phosphate concentration is sampled every 20 min and analysed using inductively coupled plasma-optical emission spectroscopy (ICP-OES, PerkinElmer, Optima 3300 DV), where the accuracy of the method is evaluated by the analysis of surface water reference material (RM) (SPS-SW2). Subsequently, the e-CCMs are further evaluated in a complex system using synthetic fresh urine. The feed cell is filled with 100 mL of urine solution, and the receiving cell is filled with 100 mL of 45 mM NaCl, 0.2 M Na_2_SO_4_ is used as the electrode solution. A membrane current density of 5 mA cm^-2^ is applied using a DC power supply (9201B, B&K Precision). The water samples are taken from the concentrated cell every 20 min, and concentrations of anions are analyzed by using Inductively Coupled Plasma Optical Emission Spectrometer (ICP-OES, for H_2_PO_4_^-^ and SO_4_^2-^) and Ion Chromatograph (IC, Dionex Aquion RFIC, for Cl^-^).

In the e-MC system, the simulated urine solution with a volume of 300 mL and pure water with a volume of 150 mL are pumped into the feed chamber and recovery chamber separately using a peristaltic pump. The e-MC is operated at a voltage of 5 V. The Na_2_SO_4_ solution is pumped into the electrode chamber. Samples are collected every 20 min, and the concentration of salt ions is measured by the Ion Chromatograph.

The phosphate permeation rate (J, mol m^-2^ h^-1^) is used to illustrate phosphate transport ability and is calculated as

$\text{J = }\frac{\text{(C}_{\text{t}}\text{-}\text{C}_{\text{0}}\text{)V}}{\text{At}}$	(6)

where $C_{t}$ and $C_{0}$ are the concentrations in the concentrated (recovery) cell at time t and time 0, V is the volume of solution, A is the membrane effective area, and t is the separation time.

The phosphate recovery rate ($\delta$, %) is calculated using equation (2):

$\text{δ = }\frac{\text{(C}_{\text{e}}\text{-}\text{C}_{\text{0}}\text{)}}{\text{C}_{\text{0}}^{\text{'}}}\text{×100}$	(7)

where $C_{e}$ and $C_{0}$ are the final and initial phosphate concentrations in the recovery chamber, $C_{0}^{'}$ is the initial phosphate concentration in the feed chamber.

The energy consumption (W, kWh mol^-1^) and current efficiency (ŋ, %) are calculated as follows:

$W=\frac{U\int_{0}^{t} Idt}{N}$	(8)

$\text{ŋ }\left( \text{\%} \right)\text{ = }\frac{\text{ZF(}\text{C}_{\text{c}}\text{V}_{\text{c}}\text{-}\text{C}_{\text{0}}\text{V}_{\text{0}}\text{)}}{\int_{\text{0}}^{\text{t}} \text{Idt}}$	(9)

where U is the voltage (V), I is the applied current (A), N is the mole number of phosphate in the concentration chamber, Z is the ionic valence, F is Faraday constant (96485 C mol^-1^). $C_{c}$ and $C_{0}$ (mol L^-1^) are the concentrations of phosphate in the recovery chamber at time t and time 0, $V_{c}$ and $V_{0}$ are the corresponding volumes of solution in the recovery chamber (L).

**Long-term stability of the membrane**

In the stability test, the synthetic urine was pumped into the dilute and concentrate chambers separately. The electrode solution of 0.2 M Na_2_SO_4_ was pumped into the electrode chamber. The experiment took 144 hours in total. Each feed solution was changed every 24 hours. For each cycle, the e-CCM was tested in electrodialysis with a single phosphate solution, and samples were collected at 20 min to calculate the phosphate ion permeation rate. The concentration of samples was quantified by an ion chromatograph (Dionex Aquion RFIC).

**Molecular dynamics simulations**

To reveal the carrier-conducting phosphate transport mechanism of e-CCM, MD simulation is performed using the large-scale Atomic/Molecular Massively Parallel Simulator (LAMMPS) software package [3]. With the LAMMPS, the SPC/E model with a diffusion coefficient close to the experimental value is adopted in the simulation of the water model, and the SHAKE algorithm is employed to maintain the rigidity of water molecules, thereby ensuring computational efficiency [4]. The simulation box is constructed with the permeation side (pure water), the feed side (water molecules and H_2_PO_4_^-^), the graphene piston, and the membrane. The Lennard-Jones (LJ) parameters for the atoms of membranes and ions are obtained from the DREIDING force field [5]. Nonbonding interactions will be calculated using standard Lennard-Jones and Coulombic potentials:

$U_{ij}=\sum4ɛ_{ij}\left[ \left( \frac{\sigma_{ij}}{r_{ij}} \right)^{12}-\left( \frac{\sigma_{ij}}{r_{ij}} \right)^{6} \right]+\sum\frac{q_{i}q_{j}}{4\piɛ_{0}r_{ij}}$	(10)

where the $ɛ_{ij}$ and $\sigma_{ij}$ represent the well depth and collision diameter, respectively. $r_{ij}$ is the distance between atom i and j, $q_{i}$ and $q_{j}$ are the electrostatic potential charges of atoms i and j, $ɛ_{0}$ is the vacuum permittivity. Cutoff distances of 1.0 nm (LJ) and 1.2 nm (Coulomb) are sequentially applied. The partical-partical-partical-mesh (PPPM) method with a tolerance of 10^-5^ is applied for the long-range electrostatic interactions [6]. Periodic boundary conditions are applied in three directions. The timestep is 1 fs. For each simulation, the energy-minimization and equilibrium simulation are firstly carried out in turn, and the temperature is adjusted to 300 K using the Nosé–Hoover thermostat, an external force of 0.1MPa is applied to each graphene piston on both sides of the model to maintain the stability of the system, followed by the production run of non-equilibrium simulation for 10 ns with an electric field of 0.015 V Å^-1^ applied on ions to mimic the e-MC conditions.

The adaptive biasing force (ABF) method is employed to calculate the potential of mean force (PMF) of ions along the direction perpendicular to the membrane plane (Z-axis). The calculation is spatially discretized with a histogram bin width of 0.05 Å. To enhance sampling efficiency, extended Lagrangian dynamics are applied with a temperature of 300 K and a fluctuation amplitude of 0.1 Å. The ABF algorithm begins to apply the biasing force after collecting 1000 samples within each interval.

**Struvite precipitation**

After the large-scale electrodialysis test, K-struvite was precipitated by adding 366.89 mg of KCl and 464.73 mg of MgCl_2_ into a 150 mL concentrated phosphate solution. The resulting K-struvite precipitate was dried at 60 °C. The atomic ratio of the precipitates was determined by XPS. The diffraction pattern obtained by XRD was analyzed to identify the crystalline phases present in the sample. The peak positions and intensities were also compared to standard reference patterns.

**Estimation of phosphate recovery from human urine**

The Food and Agriculture Organization of the United Nations (FAO) provides per capita protein consumption data on a country specific basis. This data can be used to estimate the phosphorus excreted by an individual on a country specific basis, following the method by Jönsson et al., 2004.

$phosphorus=0.011 \times(total food protein+plant food protein)$	(11)

Where the phosphorus is the total phosphorus in urine and feces in grams per capita per day (g/c/d), and both protein values have units of grams of protein per capita per day. Total food protein is the sum of protein derived from animal and plant food sources.

Plant protein contains approximately twice as much phosphorus per gram of protein as animal protein [7]. Thus, the amount of phosphorus in the excreta (urine plus feces) is obtained from the sum of animal protein intake and twice the plant protein. Equal distribution of phosphorus in urine and feces is assumed; thus, the phosphorus content in urine is 50% of the total phosphorus calculated by Eq. (11).

The most current average national total protein and vegetable protein consumption data (g/capita/day), and global phosphate fertilizer demand are all collected from FAO (as shown in detail in Data S1).

**Supplementary Figures**

## Figure S1. Schematic illustration of the global phosphorus cycle and electro-driven phosphate recovery.

**Figure S2**. Global distribution of phosphate rock reserves (million tonnes P_2_O_5_) and annual phosphate consumption (kilotons P_2_O_5_ yr^-1^) (a), Long-term average total P (TP) concentrations in surface waters over the period 2000−2022 (b).

Source data are provided in the Data S2.

Data on phosphate consumption are collected from the International Fertilizer Association. Total phosphorus ≥ 100 µg L-1 indicates hypereutrophic status [8], often associated with harmful algal blooms and oxygen depletion.

## Figure S3. Mechanism of the chemical reaction in the PP-Fh structure.

.

## Figure S4. Photographs of PP (left) and PP-Fh (right).

## Figure S5. The process of preparation of the Kevlar hydrogel membrane substrate (ANF).

## Figure S6. Schematic diagram of the structure of e-CCM, including the PP-Fh layer and ANF substrate.

## Figure S7. The cross-section SEM images of ANF/PP (a), QANF/PP (b), ANF/PP-Fh (c), and QANF/PP-Fh (d).

## **Figure S8**. The surface, zoomed-in surface, and EDX mapping images of ANF/PP (a1-a3), QANF/PP (b1-b3), ANF/PP-Fh (c1-c3), and QANF/PP-Fh (d1-d3).

## **Figure S9**. Cross-sectional TEM and EDX mapping images of ANF/PP-Fh.

## Figure S10. XRD patterns of PP-Fh and the synthesized 2-line ferrihydrite.

## **Figure S11**. XPS spectra of C 1s, N 1s, and O 1s high-resolution of ANF/PP (a1-a3), QANF/PP (b1-b3), ANF/PP-Fh (c1-c3), and QANF/PP-Fh (d1-d3).

## Figure S12. Mechanism of the chemical reaction in the QANF/PP (QANF/PP-Fh) structure.

## Figure S13. XPS spectra of the surface elements (atomic%) of QANF/PP and QANF/PP-Fh.

## Figure S14. The AFM height mapping with a scanning window of 5 μm × 5 μm of ANF/PP, ANF/PP-Fh, QANF/PP, and QANF/PP-Fh.

## Figure S15. Schematic of the electro-driven cell used for measurements of electrochemical properties.

## Figure S16. Polarization current density-voltage (C-V) curves of the AMX, ANF/PP, QANF/PP, and ANF/PP-Fh measured in 0.05 NaCl.

## Figure S17. Schematic of ion separation in electrodialysis.

## Figure S18. Phosphate concentrations in the recovery chamber. Error bars represent the standard error from triplicate measurements.

The electrodialysis was run at a current density of 5.0 mA cm^-2^, the feed and concentrated solutions were initially composed of 100 mL 40 mM phosphate with a pH = 6.

## Figure S19. XRD patterns of e-CCM after 144 hours of electrodialysis operation.

## Figure S20. Snapshots of non-equilibrium models of phosphate transport through e-CCM and non-carrier membrane at different states.

## Figure S21. Computational model of the x-z plane for PP-Fh membrane.

Colour of atoms and ions: red-oxygen, blue-nitrogen, grey-carbon, white-hydrogen, purple-Fe

## **Figure S22.** Schematic illustration of the carrier-mediated mechanism for phosphate transport.

The initial rapid phosphate diffusion is facilitated by Fe-phosphate complexation at the water/e-CCM interface. The Fe-based carrier sites in ferrihydrite act as transient binding centers that capture phosphate ions via inner-sphere Fe-O-P coordination, enabling their rapid uptake from the aqueous phase into the membrane and initiating subsequent carrier-to-carrier migration under the applied electric field.

## **Figure S23.** Arrhenius plot of 40 mM NaH_2_PO_4_ conductance through PP and PP-Fh.

Figure S24. XPS spectra for QANF/PP and QANF/PP-Fh after electrodialysis with 40 mM P solution. **(a)** Wide scan XPS spectra for the QANF/PP+P and QANF/PP-Fh+P membranes; **(b)** high-resolution P 2p XPS spectra for the QANF/PP-Fh+P membrane; **(c)** high-resolution Fe 2p XPS spectra for the pristine QANF/PP-Fh and QANF/PP-Fh+P; **(d)** high-resolution O 1s XPS spectra for the pristine QANF/PP-Fh and QANF/PP-Fh+P.

To verify the chemical interactions between phosphate and ferrihydrite in the e-CCM after electrodialysis, XPS characterization was conducted. Following phosphate permeation, XPS analysis detected approximately 1.2% P in QANF/PP-Fh, whereas no phosphorus signal was observed in QANF/PP. The high-resolution P 2p spectrum of QANF/PP-Fh+P exhibits a new peak at 132.6 eV, corresponding to P-O groups [9]. Moreover, after phosphate adsorption, the binding energies of Fe 2p and O 1s in QANF/PP-Fh shift relative to those in the pristine membrane, which are attributed to the chemical interaction of Fe-O-P [10,11]. These XPS results of P 2p, Fe 2p, and O 1s spectra provide clear spectroscopic evidence of phosphate sorption onto Fh within the membrane, elucidating the enhanced phosphate transport observed in the e-CCM.

## **Figure S25.** Photos of AMX (left) and CEM (right).

## **Figure S26.** Photo of the membrane assembled in the electrodialysis stack.

## **Figure S27.** Polarization current density-voltage (C-V) curves of the QANF/PP-Fh measured in simulated urine.

## **Figure S28.** Temporal evolution of anions in the feed and recovery chambers of AMX under an optimized voltage of 5 V.

Error bars represent the standard error from duplicate measurements.

## **Figure S29.** Temporal evolution of phosphate in the feed and recovery chambers of AMX and QANF/PP-Fh. Error bars represent the standard error from duplicate measurements.

## **Figure S30.** SEM images of K-struvite and element mapping of Mg and K distribution.

Figure S31. Comparison in phosphate recovery rate of AMX and QANF/PP-Fh membranes in e-MC. Error bars represent the standard error from duplicate measurements.

# Supplementary Tables

## Table S1. Names of the prepared membranes.

| Name | Fe in casting solution (wt%) | ETAC content (wt%) |
| --- | --- | --- |
| ANF/PP | 0 | 0 |
| QANF/PP | 0 | 1 |
| ANF/PP-Fh | 1.08% | 0 |
| QANF/PP-Fh | 1.08% | 1 |

## Table S2. The recipe for preparing a fresh urine solution.

| Chemical reagent | Concentration (g/L) | Theoretical concentrations (mM) | |
| --- | --- | --- | --- |
|  |  | Cation concentration | Anion concentration |
| MgCl_2_•6H_2_O | 0.43 | Mg^2+^ (2.115) | Cl^-^ (4.230) |
| CaCl_2_ | 0.19 | Ca^2+^ (1.768) | Cl^-^ (3.537) |
| Na₂SO₄ | 1.5 | Na^+^ (21.121) | SO₄^2-^ (10.560) |
| NaCl | 2.5 | Na^+^ (42.779) | Cl^-^ (42.779) |
| NaH_2_PO_4_•2H_2_O | 3.2 | Na^+^ (20.512) | H_x_PO_4_^(3-x)^ (20.512) |
| KCl | 1.5 | K^+^ (20.121) | Cl^-^ (20.121) |
| Urea | 15 | / | / |

## Table S3. Comparison of the phosphate separation performance of electro-driven membranes in the electrodialysis system in the literature.

| IEMs | Current density (mA cm^-2^) | P concentration (mmol L^-1^) | Phosphate permeation rate  (mol∙m^-2^∙h^-1^) | Phosphate recovery rate (%) | Energy consumption  (kwh mol^-1^ P) | Current efficiency % | Ref |
| --- | --- | --- | --- | --- | --- | --- | --- |
| Neosepta AMX |  | 3.22 | 0.0706 | 65.9 | 2.87 | 75 | [12] |
| JAM-II-07 | 10 | 6.45 | 0.042 | 94.4 | 20.1 |  | [13] |
|  | 12.5 | 6.45 | 0.068 | 90.3 | 12.4 |  |  |
|  | 15 | 6.45 | 0.098 | 86.1 | 5.7 |  |  |
| HDX200 | 0.5 | 0.13 | 0.013 | 86.8 |  |  | [14] |
| Ralex AMH-PES | 0.36 | 7.74 |  | 73.9 |  |  | [15] |
| MemBrain AM | 16.7 | 15.88 | 0.055 | 47.3 |  |  | [16] |
| AM (PC-SA) | 3.125 | 8 | 0.2009 | 35.5 |  |  | [17] |
|  | 3.125 | 2.75 | 0.024 | 51.9 | 2.17 |  |  |
| PC-SA | 4.69 | 3 | 0.1505 | 53 |  | 8.3-10.6 | [18] |
| PC-SA | 6.25 | 5 | 0.1823 | 42 |  | 10.2-17 |  |
| PC-SA | 4.69 | 7 | 0.4167 | 68.6 |  | 21.3-26.6 |  |
| Neosepta AMX | 0.3 | 0.32 | 0.0011 | 70 | 13.6 |  | [19] |
|  | 0.31 | 0.32 | 0.0016 | 84.8 | 22.9 |  |  |
|  | 0.59 | 0.32 | 0.0013 | 50 | 54.8 |  |  |
| PC-SA | 5 | 1.29 | 0.0252 | 73 | 2.7 |  | [20] |
| PC-SA | 4.53 | 1.29 | 0.015 | 50 | 3.2 |  |  |
| PE AEM | 7.8 | 6.4 | 0.259 | 90 | 1.3 |  | [21] |
| MemBrain | 2.9 | 21.9 | 0.139 | 70 | 6.3 | 10 | [22] |
| MemBrain | 2.9 | 18.7 | 0.127 | 53.9 |  |  |  |
| PC-SA | 0.78 | 0.75 | 0.031 | 40 |  |  | [23] |
| PC-SA | 0.94 | 1.64 | 0.11 | 50 |  |  |  |
| HMO-CEM |  |  | 0.0014 | 1 |  |  | [11] |
| e-CCM | 5 | 40 | 0.92 | 98.4 | 0.19 | 91.3 | this work |

## Table S4. The measured ion concentration in the prepared fresh urine.

| Composition | Measured concentration (mM) |
| --- | --- |
| Mg^2+^ | 2.287 |
| Ca^2+^ | 1.786 |
| Na^+^ | 92.593 |
| K^+^ | 20.314 |
| SO₄^2-^ | 11.584 |
| H_x_PO_4_ ^(3-x)^ | 20.305 |
| Cl^-^ | 66.742 |

All the elements were measured with ICP-OES, except Cl^-^ with Ion Chromatography (IC).

## Table S5. The hydrated diameters of anions [24]

| Metal ion | Hydrated diameter (Å) | Hydration energy (kJ mol^-1^) |
| --- | --- | --- |
| Cl^-^ | 6.64 | -367 |
| SO_4_^2-^ | 7.58 | -1035 |
| H_2_PO_4_^-^ | 6.78 | -1170 |

## Table S6. Fe concentrations in the recovery chamber over 144 hours of electrodialysis operation.

| Time (h) | Fe 238.204 {141} (Axial) (mg L^-1^)  Limit of Quantification (0.003) |
| --- | --- |
| 0 | -0.000217272 |
| 12 | -0.000290178 |
| 24 | -0.000352427 |
| 36 | -0.000106577 |
| 48 | -0.000145673 |
| 60 | 0.00012024 |
| 72 | 2.52389E-05 |
| 84 | 6.31947E-05 |
| 96 | 0.000155162 |
| 108 | 7.88334E-05 |
| 120 | 0.000120073 |
| 132 | 3.79001E-05 |
| 144 | 0.000144115 |

The Fe concentration was measured using inductively coupled plasma-optical emission spectroscopy (ICP-OES, PerkinElmer, Optima 3300 DV).

## Table S7. Pore structure parameters and porosity of PP and PP-Fh membranes based on Zeo++ analysis [25].

|  | PP | PP-Fh |
| --- | --- | --- |
| Global pore Diameter (Å) | 14.61 | 14.61 |
| Maximum Pore Diameter (Å) | 14.58 | 14.61 |
| Pore Limiting Diameter (Å) | 10.50 | 10.50 |
| Porosity | 0.46 | 0.44 |

## Table S8. The economic analysis of phosphate recovery

|  | e-CCM | AMX |
| --- | --- | --- |
| **Operation conditions** | 5 V | |
| **Energy cost** |  |  |
| Sp(kg/Kw∙h) | 0.34 | 0.26 |
| K-struvite recovered per year(kg/y) | 5.27 | 4.59 |
| Electricity price ($/Kw∙h) | 0.1 | 0.1 |
| Energy cost for K-struvite recovery ($/kg) | 0.297 | 0.379 |
| Energy cost for peripheral equipment ($/kg) | 0.01485 | 0.01895 |
| Total energy cost ($/kg) | 0.31 | 0.40 |
| **Investment cost** |  |  |
| Lifetime of the entire device (y) | 3 | 3 |
|  |  |  |
| PAN ($) | 0.28 |  |
| DMSO ($) | 0.78 |  |
| PEI ($) | 0.19 |  |
| FeCl_3_ ($) | 0.02 |  |
| ETAC ($) | 0.06 |  |
| KANF $ | 0.03 |  |
| KOH $ | 0.005 |  |
| Commercial IEMs ($) | 10.89^a^ |  |
| Total membrane and chemicals cost ($) | 12.26 | 14.53 |
|  |  |  |
| Membrane equipment cost ($) | 18.39 | 21.79 |
| Auxiliary equipment cost ($) | 12.87 | 15.26 |
| Total investment cost ($) | 31.26 | 37.06 |
| Amortization per year ($/y) | 12.13 | 14.38 |
| Maintenance cost per year ($/y) | 3.13 | 3.71 |
| Fixed cost per year ($/y) | 15.25 | 18.08 |
| Fixed cost for unit K-struvite recovery ($/kg) | 2.90 | 3.94 |
| **Overall recovery cost ($/kg)** | 3.21 | 4.34 |

The prices of chemical products are based on Sigma-Aldrich prices and the amount needed to cast membranes with dimensions of 11 cm × 11 cm.

^a^Three commercial membranes, including two CMX and one AMX, were used in the e-MC system with e-CCM.

## Table S9. Characteristics of the FUJIFILM ion exchange membranes

|  | Type | Ion exchange capacity (mmol·g^-1^) | Surface resistance (Ω·cm^2^) | Thickness (μm) |
| --- | --- | --- | --- | --- |
| AMX | Type-I normal anion exchange membrane | 1.7 | 1.3 | 125 |
| CEM | Standard cation exchange membrane | 1.0 | 2.7 | 135 |

## Movie S1.

## Molecular dynamics trajectory animation of e-CCM.

## Movie S2.

## Molecular dynamics trajectory animation of non-carrier membrane.

## Data S1.

## Average national total protein and vegetable protein consumption data and global phosphate fertilizer consumption data.

## Data S2.

## Global distribution of phosphate rock reserves, annual phosphate consumption, and long-term average total P concentrations in surface waters.

## Data S3.

## The economic analysis of phosphate recovery.

# References

1. X. Wang, Z. Jiang, J. Qian, W. Fu, B. Pan, “Structure Evolution of Iron (Hydr)oxides under Nanoconfinement and Its Implication for Water Treatment,” *Environmental Science & Technology* 58 (2024): 826–835, <https://doi.org/10.1021/acs.est.3c05760>.

2. Y. Lan, D. Zhou, L. Lai, H. Qi, L. Xia, S. Depuydt, B. Van der Bruggen, Y. Zhao, “A Monovalent Selective Anion Exchange Membrane Made by Poly(2,6-dimethyl-1,4-phenyl oxide) for Bromide Recovery,” *Separation and Purification Technology* 305 (2023): 122377, <https://doi.org/10.1016/j.seppur.2022.122377>.

3. A.P. Thompson, H.M. Aktulga, R. Berger, D.S. Bolintineanu, W.M. Brown, P.S. Crozier, P.J. in ’t Veld, A. Kohlmeyer, S.G. Moore, T.D. Nguyen, R. Shan, M.J. Stevens, J. Tranchida, C. Trott, S.J. Plimpton, “LAMMPS - A Flexible Simulation Tool for Particle-based Materials Modeling at the Atomic, Meso, and Continuum Scales,” *Computer Physics Communications* 271 (2022): 108171, <https://doi.org/10.1016/j.cpc.2021.108171>.

4. R. Qiu, J. Xiao, X.D. Chen, C. Selomulya, X. Zhang, M.W. Woo, “Comparison of the Effects of Edge Functionalized Graphene Oxide Membranes on Monovalent Cation Selectivity,” *Journal of Membrane Science* 620 (2021): 118892, <https://doi.org/10.1016/j.memsci.2020.118892>.

5. S.L. Mayo, B.D. Olafson, W.A. Goddard, “DREIDING: A Generic Force Field for Molecular Simulations,” *Journal of Physical Chemistry* 94 (2002): 8897–8909, <https://doi.org/10.1021/j100389a010>.

6. F. Xu, M. Wei, X. Zhang, Y. Wang, “Effect of Hydrophilicity on Water Transport through Sub-Nanometer Pores, *Journal of Membrane Science* 611 (2020): 118297, <https://doi.org/10.1016/j.memsci.2020.118297>.

7. H. Jönsson, A.R. Stintzing, E. Salomon, “Guidelines on the Use of Urine and Faeces in Crop Production i Guidelines on the Use of Urine and Faeces in Crop Production,” 2004. [www.ecosanres.org](http://www.ecosanres.org).

8. D. Naderian, R. Noori, E. Heggy, S.M. Bateni, R. Bhattarai, A. Nohegar, S. Sharma, “A Water Quality Database for Global Lakes,” *Resources, Conservation and Recycling* 202 (2024): 107401, <https://doi.org/10.1016/j.resconrec.2023.107401>.

9. X. Chen, X. Ding, C. Wang, Z. Feng, L. Xu, X. Gao, Y. Zhai, D. Wang, “A Multi-shelled CoP Nanosphere Modified Separator for Highly Efficient Li-S Batteries,” *Nanoscale* 10 (2018): 13694–13701, <https://doi.org/10.1039/c8nr03854f>.

10. W. Xu, W. Zheng, F. Wang, Q. Xiong, X.L. Shi, Y.K. Kalkhajeh, G. Xu, H. Gao, “Using Iron Ion-loaded Aminated Polyacrylonitrile Fiber to Efficiently Remove Wastewater Phosphate,” *Chemical Engineering Journal* 403 (2021): 126349, <https://doi.org/10.1016/j.cej.2020.126349>.

11. A. Iddya, P. Zarzycki, R. Kingsbury, C.M. Khor, S. Ma, J. Wang, I. Wheeldon, Z.J. Ren, E.M. V Hoek, D. Jassby, “A Reverse-selective Ion Exchange Membrane for the Selective Transport of Phosphates via an Outer-sphere Complexation-diffusion Pathway,” *Nature Nanotechnology* 17 (2022):1222–1228, <https://doi.org/10.1038/s41565-022-01209-x>.

12. X. Wang, Y. Wang, X. Zhang, H. Feng, C. Li, T. Xu, “Phosphate Recovery from Excess Sludge by Conventional Electrodialysis (CED) and Electrodialysis with Bipolar Membranes (EDBM),” *Industrial & Engineering Chemistry Research* 52 (2013): 15896–15904, <https://doi.org/10.1021/ie4014088>.

13. X. Wang, X. Zhang, Y. Wang, Y. Du, H. Feng, T. Xu, “Simultaneous Recovery of Ammonium and Phosphorus via the Integration of Electrodialysis with Struvite Reactor,” *Journal of Membrane Science* 490 (2015): 65–71, <https://doi.org/10.1016/j.memsci.2015.04.034>.

14. E.H. Rotta, C.S. Bitencourt, L. Marder, A.M. Bernardes, “Phosphorus Recovery from Low Phosphate-containing Solution by Electrodialysis,” *Journal of Membrane Science* 573 (2019): 293–300, <https://doi.org/10.1016/j.memsci.2018.12.020>.

15. W. Pronk, M. Biebow, M. Boller, “Treatment of Source-separated Urine by A Combination of Bipolar Electrodialysis and a Gas Transfer Membrane,” *Water Science and Technology* 53 (2006): 139–146, <https://doi.org/10.2166/wst.2006.086>.

16. L. Shi, S. Xie, Z. Hu, G. Wu, L. Morrison, P. Croot, H. Hu, X. Zhan, “Nutrient Recovery from Pig Manure Digestate using Electrodialysis Reversal: Membrane Fouling and Feasibility of Long-term Operation,” *Journal of Membrane Science* 573 (2019): 560–569, <https://doi.org/10.1016/j.memsci.2018.12.037>.

17. Y. Zhang, E. Desmidt, A. Van Looveren, L. Pinoy, B. Meesschaert, B. Van Der Bruggen, “Phosphate Separation and Recovery from Wastewater by Novel Electrodialysis,” *Environmental Science & Technology* 47 (2013): 5888–5895, <https://doi.org/10.1021/es4004476>.

18. A.T.K. Tran, Y. Zhang, D. De Corte, J.B. Hannes, W. Ye, P. Mondal, N. Jullok, B. Meesschaert, L. Pinoy, B. Van Der Bruggen, “P-recovery as Calcium Phosphate from Wastewater using an Integrated Selectrodialysis/Crystallization Process,” *Journal of Cleaner Production* 77 (2014) 140–151. <https://doi.org/10.1016/j.jclepro.2014.01.069>.

19. R. Liu, Y. Wang, G. Wu, J. Luo, S. Wang, “Development of a Selective Electrodialysis for Nutrient Recovery and Desalination during Secondary Effluent Treatment,” *Chemical Engineering Journal* 322 (2017): 224–233, <https://doi.org/10.1016/j.cej.2017.03.149>.

20. Z.L. Ye, K. Ghyselbrecht, A. Monballiu, L. Pinoy, B. Meesschaert, “Fractionating Various Nutrient Ions for Resource Recovery from Swine Wastewater using Simultaneous Anionic and Cationic Selective-Electrodialysis,” *Water Research* 160 (2019): 424–434, <https://doi.org/10.1016/j.watres.2019.05.085>.

21. K.C. Kedwell, M.K. Jørgensen, C.A. Quist-Jensen, T.D. Pham, B. Van der Bruggen, M.L. Christensen, “Selective Electrodialysis for Simultaneous but Separate Phosphate and Ammonium Recovery,” *Environmental Technology* 42 (2021): 2177–2186. <https://doi.org/10.1080/09593330.2019.1696410>.

22. L. Shi, Y. Hu, S. Xie, G. Wu, Z. Hu, X. Zhan, “Recovery of Nutrients and Volatile Fatty Acids from Pig Manure Hydrolysate using Two-stage Bipolar Membrane Electrodialysis,” *Chemical Engineering Journal* 334 (2018): 134–142, <https://doi.org/10.1016/j.cej.2017.10.010>.

23. J. De Paepe, R.E.F. Lindeboom, M. Vanoppen, K. De Paepe, D. Demey, W. Coessens, B. Lamaze, A.R.D. Verliefde, P. Clauwaert, S.E. Vlaeminck, “Refinery and Concentration of Nutrients from Urine with Electrodialysis Enabled by Upstream Precipitation and Nitrification,” *Water Research* 144 (2018): 76–86, <https://doi.org/10.1016/j.watres.2018.07.016>.

24. Zhao, N. Mamrol, W.A. Tarpeh, X. Yang, C. Gao, B. Van der Bruggen, “Advanced Ion Transfer Materials in Electro-driven Membrane Processes for Sustainable Ion-resource Extraction and Recovery,” *Progress in Materials Science* 128 (2022): 100958, <https://doi.org/10.1016/j.pmatsci.2022.100958>.

25. T.F. Willems, C.H. Rycroft, M. Kazi, J.C. Meza, M. Haranczyk, “Algorithms and Tools for High-Throughput Geometry-based Analysis of Crystalline Porous Materials,” *Microporous and Mesoporous Materials* 149 (2012): 134–141, <https://doi.org/10.1016/j.micromeso.2011.08.020>.
